# In silico assessment on TdP risks of drug combinations under CiPA paradigm

**DOI:** 10.1038/s41598-023-29208-5

**Published:** 2023-02-20

**Authors:** Ali Ikhsanul Qauli, Aroli Marcellinus, Muhammad Aldo Setiawan, Andi Faiz Naufal Zain, Azka Muhammad Pinandito, Ki Moo Lim

**Affiliations:** 1 Robotics and Artificial Intelligence Engineering, Faculty of Advanced Technology and Multidiscipline, Universitas Airlangga, Surabaya, Indonesia; 2 IT Convergence Engineering, Kumoh National Institute of Technology, Gumi, 39177 Republic of Korea; 3 Medical IT Convergence Engineering, Kumoh National Institute of Technology, Gumi, 39253 Republic of Korea; 4 Meta Heart Inc., Gumi, 39253 Republic of Korea

**Keywords:** Virtual screening, Virtual drug screening

## Abstract

Researchers have recently proposed the Comprehensive *In-vitro* Proarrhythmia Assay (CiPA) to analyze medicines’ TdP risks. Using the TdP metric known as qNet, numerous single-drug effects have been studied to classify the medications as low, intermediate, and high-risk. Furthermore, multiple medication therapies are recognized as a potential method for curing patients, mainly when limited drugs are available. This work expands the TdP risk assessment of drugs by introducing a CiPA-based in silico analysis of the TdP risk of combined drugs. The cardiac cell model was simulated using the population of models approach incorporating drug-drug interactions (DDIs) models on several ion channels for various drug pairs. Action potential duration (APD90), qNet, and calcium duration (CaD90) were computed and analyzed as biomarker features. The drug combination maps were also used to illustrate combined medicines' TdP risk. We found that the combined drugs alter cell responses in terms of biomarkers such as APD90, qNet, and CaD90 in a highly nonlinear manner. The results also revealed that combinations of high-risk with low-risk and intermediate-risk with low-risk drugs could result in compounds with varying TdP risks depending on the drug concentrations.

## Introduction

Torsade de pointes (TdP) is an abnormality of the heart that can cause sudden cardiac death. Several medications have been withdrawn from the market due to drug-induced TdP^1^, which is now a significant concern for the pharmaceutical industry and international regulatory agencies. Researchers have recently proposed a new cardiac safety paradigm for assessing drug-induced TdP, the Comprehensive In-vitro Proarrhythmia Assay (CiPA), which includes the in-silico simulation of TdP risk of drugs^2^ to the assessment procedure. Numerous studies incorporating mechanistic in-silico under the CiPA paradigm have been successfully published. An early study^3^ suggested using multiple ion channel inhibitions to predict the TdP risk of drugs. The authors applied the drug’s concentration and 50% inhibitory concentration (IC50) under Hill’s model^4^ to the human Ether-à-go-go-related gene (hERG), sodium (Na), and L-type calcium (CaL).

Further research^5–7^ incorporated the blocking effects of drugs on multiple ion channels of the human ventricular tissue model proposed by O’Hara et al.^8^ by introducing dynamic inhibition effects to the hERG channel. In addition, other research by Passini et al.^9,10^ demonstrated the predictive ability of in-silico assessment of TdP risk of drugs by employing calibrated human ventricular models derived from experimental data^11–13^. Passini et al. discovered that in-silico trials could predict the TdP risk of pharmaceuticals with an accuracy of 89%. Moreover, when combined with features such as the electromechanical window, the prediction performance can reach 90% with ten times the therapeutic concentrations (EFTPCmax). In contrast, its counterpart with repolarization anomaly features can only achieve the same level of precision with 100 EFTPCmax.

Despite the excellent performance demonstrated by the earlier studies, most studies focus on single-drug effects without assessing combined drugs’ effects. During medical treatment, patients are frequently administered multiple medications in clinical practice. The use of polypharmacy is intended to maximize the treatment’s efficacy while minimizing adverse effects. However, polypharmacy may generate different effects than its single-drug counterpart, and the unknown drug combinations’ effects may result in suboptimal, if not dangerous, patient care. Consequently, healthcare agencies such as the European Medicines Agency (EMA) already recommend pharmacodynamics (PD) interaction studies when multiple medications compete for the same target and are likely to be administered concurrently, such as QT-prolonging drugs^14^. Furthermore, several studies have reported the effects of drug-drug interactions (DDIs) on antiarrhythmic drugs when combined with antibiotics, antipsychotics, antiallergic agents, and prokinetic agents^15–17^. As the CiPA paradigm has already proven to be quite effective in predicting the risks of single drug effects, it can be extended to include the study of assessing the TdP risk of the polypharmacy effects to comprehend the unidentified multiple drug effects better.

The study of DDIs to evaluate the PD effects is numerous, and the dose-response effect becomes an essential part of studying PD effects. Among the early studies on DDIs were reported by ^18,19^. However, when dose-response data is unavailable, such a model may be unhelpful^20^. As we advance to recent studies, the current methodological landscape includes: (i) graphical techniques such as the isobologram method^21^, the fractional inhibitory concentration (FIC)^22^ indices or combination index^23^, and (ii) response surface method^24^. Furthermore, a study from Wicha et al.^25^ proposed general pharmacodynamic interaction (GPDI) that was validated with 200 combination experiments in Saccharomyces cerevisiae reported by Cokol et al.^26^. The authors found that the majority (67%) of the combination experiments were monodirectional interactions that previous method such as isobole analysis^21,26^ and Greco et al.^27^ model could not classify.

Some researchers have reported using the DDIs model to predict the TdP risk of polypharmacy. Wiśniowska et al.^28^ evaluated DDIs at the hERG channel under the CiPA paradigm and compared experimental measurements of the inhibition effects of combined drugs with theoretical models. They discovered that the allotopic model provided the best empirical fit for predicting combination effects incorporating loratadine, desloratadine, and ketoconazole. Another study by Delaunois et al.^29^ reported using the drug combination model to predict the TdP risks associated with the antimalarial drugs during the first wave of the COVID-19 pandemic. DDIs data (dose-response) effects were measured experimentally at the hERG channel. The combined drug effects on human ventricular cells were predicted and evaluated using a simple DDIs (additive) model for multiple ion channels. A study from Varshneya et al.^30^ combined pharmacokinetics (PK) with quantitative system pharmacology (QSP) to assess the effects of four drugs (drugs proposed to treat COVID-19: lopinavir, ritonavir, chloroquine, and azithromycin) on cardiac electrophysiology. From statistical analysis of population of models, the authors found that women with heart failure are especially susceptible to arrhythmias. Another study by Whittaker et al.^31^ assessed the TdP risk of hydroxychloroquine, chloroquine, and other QT-prolonging drugs. The authors predicted that the combination of moxifloxacin with hydroxychloroquine possessed a higher TdP risk than hydroxychloroquine alone. Another study from Montnach et al.^32^ assessed the TdP risk of hydroxychloroquine, azithromycin, and other drugs such as mexiletine, dofetilide, and quinidine under clinical factors such as tachycardia, hypokalaemia, and subclinical to mild long QT syndrome. One of their findings was that mexiletine minimized the harmful effects of azithromycin and hydroxychloroquine in subclinical settings.

However, some experimental studies of combined drugs’ effect (dose-response data) show no interactions between drugs that may cause difficulty in assessing the TdP risks of combined drugs. For example, the experiment done by Delaunois et al.^29^ showed that the combined azithromycin and hydroxychloroquine resulted in no synergistic or antagonistic interaction. Furthermore, a study conducted by Wiśniowska et al.^28^ incorporating several combinations of loratadine, desloratadine, and ketoconazole predicted straight-line effects on the isobologram graph, indicating no synergism and antagonism. Further experimental studies may be required to assess the impact of drug combinations that show DDIs (synergism or antagonism). However, albeit the scarcity of drug combination experiments, especially for TdP risk assessment, a typical way to solve the lack of experimental data is by incorporating DDIs models that require only single drug information, such as the allotopic and syntopic models^33^. The Allotopic model is essentially the same as the Bliss independence model^34–37^. In contrast, the syntopic model is analogous to Loewe Additivity (LA) model, as LA’s original definition requires a mutual maximum effect to exist^19^. In this study, authors utilize allotopic and syntopic models to assess the TdP risk of multiple medications on a model of human cardiac cells, extending the analysis to include several doses of two-drug combinations.

## Methods

This section briefly reviews the cardiac cell model used in the simulation. Furthermore, the combined drugs’ effects based on the allotopic and syntopic models, as well as the simulation protocol to obtain features such as APD90, CaD90, and qNet are also described in this section.

### The cardiac cell model

The cell model deployed in the simulation was initially based on the undiseased human cardiac cell model proposed by O’Hara et al.^8^ that was later extended and optimized by Li et al.^7^ and Dutta et al.^6^. The membrane potential $\left({\mathrm{V}}_{\mathrm{m}}\right)$ of the cardiac cell is expressed in the mathematical formula as follows:

$$\frac{{{\text{dV}}_{{\text{m}}} }}{{{\text{dt}}}} = - \frac{1}{{{\text{C}}_{{\text{m}}} }}\left( {{\text{I}}_{{{\text{ion}}}} + {\text{I}}_{{{\text{stim}}}} } \right)$$

where the ${\mathrm{C}}_{\mathrm{m}}$ is the membrane capacitance, ${\mathrm{I}}_{\mathrm{stim}}$ is the stimulus current, and ${\mathrm{I}}_{\mathrm{ion}}$ is the sum of ionic transmembrane currents. The corresponding ionic currents are the sodium current $\left({\mathrm{I}}_{\mathrm{Na}}\right)$, transient outward potassium current $\left({\mathrm{I}}_{\mathrm{to}}\right)$, L-type calcium current $\left({\mathrm{I}}_{\mathrm{CaL}}\right)$, sodium current through L-type calcium channel $\left({\mathrm{I}}_{\mathrm{CaNa}}\right)$, potassium current through L-type calcium channel $\left({\mathrm{I}}_{\mathrm{CaK}}\right)$, rapid delayed rectifier potassium current $\left({\mathrm{I}}_{\mathrm{Kr}}\right)$, slow delayed rectifier potassium current $\left({\mathrm{I}}_{\mathrm{Ks}}\right)$, inward rectifier potassium current $\left({\mathrm{I}}_{\mathrm{K}1}\right)$, sodium-calcium exchange current $\left({\mathrm{I}}_{\mathrm{NaCa}}\right)$, sodium–potassium ATPase current $\left({\mathrm{I}}_{\mathrm{NaK}}\right)$, background currents $\left({\mathrm{I}}_{\mathrm{Nab}},{\mathrm{I}}_{\mathrm{Cab}},{\mathrm{I}}_{\mathrm{Kb}}\right)$, and sarcolemma calcium pump current $\left({\mathrm{I}}_{\mathrm{pCa}}\right)$.

For incorporating drug inhibition effects during drugs assessment under CiPA paradigm, Mirams et al.^3^ proposed the model for the inhibition effects of a single drug on an ion channel by using a conductance-block formulation as follows:

$${\text{g}}_{{\text{i}}} = {\text{g}}_{{{\text{control}},{\text{i}}}} \left[ {1 + \left( {\frac{{\left[ {\text{D}} \right]}}{{\left[ {{\text{IC}}_{50} } \right]}}} \right)^{{\text{n}}} } \right]^{ - 1}$$

where ${\mathrm{g}}_{\mathrm{control},\mathrm{i}}$ is the maximum conductance of channel $\mathrm{i}$, $\left[{\mathrm{IC}}_{50}\right]$ is the inhibitory concentration 50%, $\left[\mathrm{D}\right]$ is the drug concentration, and $\mathrm{n}$ is the Hill coefficient. The value of $\left[{\mathrm{IC}}_{50}\right]$ and $\mathrm{n}$ can be obtained from fitting the dose–response experiment for each ion channel to the conductance-block model.

Furthermore, Li et al.^7^ extended the model proposed by O’hara et al.^8^ and Mirams et al.^3^ by including the dynamic drug-hERG interactions model on ${\mathrm{I}}_{\mathrm{Kr}}$ and put a revised scaling factor on the maximum conductance of ${\mathrm{I}}_{\mathrm{Kr}}$ to better replicate the behavior of ${\mathrm{I}}_{\mathrm{Kr}}$ from experimental data. Moreover, Dutta et al.^6^ provided some optimization on the scaling factor of maximum conductance of ${\mathrm{I}}_{\mathrm{Kr}}$, ${\mathrm{I}}_{\mathrm{Ks}}$, ${\mathrm{I}}_{\mathrm{K}1}$, ${\mathrm{I}}_{\mathrm{CaL}}$, and ${\mathrm{I}}_{\mathrm{NaL}}$ based on the model proposed by Li et al.^7^. The optimized scaling factors were introduced to allow a better fit to the APD rate dependence experimental data under control and drug blocking situation. We deployed the model proposed by Dutta et al.^6^ with only the conductance-block model without dynamic characteristics of ${\mathrm{I}}_{\mathrm{Kr}}$ under drug effects. Detailed formulas for ion channels, membrane potentials, and others can be seen in ^6–8^ and references therein.

### Drug-drug interactions model

In this study, the ion channel inhibition effects of combined drugs were modeled by using DDIs model of allotopic and syntopic models proposed by Jarvis et al.^33^. The allotopic model is defined based on the idea that pharmacological effects are the results of probabilistic processes and that drugs work independently so that their different sites of action prevent them from interfering with one another while yet contributing to a common outcome^34^. Suppose that two drugs $\mathrm{A}$ and $\mathrm{B}$ have inhibition effects ${\mathrm{E}}_{\mathrm{A},\mathrm{i}}$ and ${\mathrm{E}}_{\mathrm{B},\mathrm{i}}$ on channel $\mathrm{i}$. The probabilistic combined effect under the allotopic model $\left({\mathrm{E}}_{\mathrm{AB},\mathrm{i}}\right)$ can be expressed as follow:

$${\text{E}}_{{{\text{AB}},{\text{i}}}} = {\text{E}}_{{{\text{A}},{\text{i}}}} + {\text{E}}_{{{\text{B}},{\text{i}}}} - {\text{E}}_{{{\text{A}},{\text{i}}}} \times {\text{E}}_{{{\text{B}},{\text{i}}}}$$

where the $0\le {\mathrm{E}}_{\mathrm{A},\mathrm{i}}\le 1$ and $0\le {\mathrm{E}}_{\mathrm{B},\mathrm{i}}\le 1$. Each single drug effect ${\mathrm{E}}_{\mathrm{A},\mathrm{i}}$ or ${\mathrm{E}}_{\mathrm{B},\mathrm{i}}$ is formulated by the conductance-block model.

Furthermore, in contrast to the allotopic model that the two drugs have different binding sites, the two drugs in the syntopic model share the same binding site so that while one drug binds, the other cannot. The syntopic model is designed based on competitive interactions^35,36^. The combined effect under the syntopic model $\left({\mathrm{E}}_{\mathrm{AB},\mathrm{i}}\right)$ can be expressed as follows:

$${\text{E}}_{{{\text{AB}},{\text{i}}}} = \frac{{{\text{E}}_{{{\text{A}},{\text{i}}}} + {\text{E}}_{{{\text{B}},{\text{i}}}} - 2 \times {\text{E}}_{{{\text{A}},{\text{i}}}} \times {\text{E}}_{{{\text{B}},{\text{i}}}} }}{{1 - {\text{E}}_{{{\text{A}},{\text{i}}}} \times {\text{E}}_{{{\text{B}},{\text{i}}}} }}$$

where the definition for ${\mathrm{E}}_{\mathrm{A},\mathrm{i}}$ and ${\mathrm{E}}_{\mathrm{B},\mathrm{i}}$ are the same as in the allotopic model.

### Simulation protocols

The simulation protocol to obtain biomarker features such as APD90, CaD90 and qNet is as follows: First, the data of ${\mathrm{IC}}_{50}$ and Hill coefficient $\mathrm{n}$ for 12 CiPA drugs need to be provided. Seven ion channels are incorporated under the drug effects: $\mathrm{CaL}$, $\mathrm{K}1$, $\mathrm{Ks}$, $\mathrm{Na}$, $\mathrm{NaL}$, $\mathrm{to}$, and $\mathrm{hERG}$ or $\mathrm{Kr}$. The data of ${\mathrm{IC}}_{50}$ and $\mathrm{n}$ are fitted from dose–response experimental data and bootstrapped to obtain 100 samples using Markov chain Monte Carlo (MCMC) simulation as proposed by Chang et al.^5^. The data and scripts for getting samples of ${\mathrm{IC}}_{50}$ and $\mathrm{n}$ of 12 CiPA drugs can be found on the web at https://github.com/FDA/CiPA.

Furthermore, the combined drugs’ effects are deployed using the allotopic and syntopic models for the seven ion channels. For each sample, the drug simulation was conducted by following the simulation protocol proposed by Chang et al.^5^: the model is initialized by simulating 1,000 pacing to obtain a steady state of control or drug-free simulation. Then the steady-state values of the model are used, combined with the combined drugs’ effects on seven ion channels for 1,000 beats. Each beat for both drug-free and drug effect simulation last for 2,000 ms. Out of the last 250 beats, the action potential with the steepest repolarization gradient $\left(\mathrm{dV}/{\mathrm{dt}}_{\mathrm{repol}}\right)$ is considered for calculating features such as APD90, qNet, and CaD90. Steepest $\mathrm{dV}/{\mathrm{dt}}_{\mathrm{repol}}$ is defined as the maximum change in membrane potential per unit time between 30 to 90% for the fully repolarized AP. If the AP is repolarized by 30% but did not reach 90%, the steepest $\mathrm{dV}/{\mathrm{dt}}_{\mathrm{repol}}$ is calculated between 30% repolarization until the end of the single beat period $(\mathrm{t}=\mathrm{2,000 ms})$. APD90 is the action potential duration calculated from the peak of action potential (AP) to 90% of its value. The qNet is the total net charge calculated from six ionic currents $\left({\mathrm{I}}_{\mathrm{Kr}},{\mathrm{I}}_{\mathrm{CaL}},{\mathrm{I}}_{\mathrm{NaL}},{\mathrm{I}}_{\mathrm{to}},{\mathrm{I}}_{\mathrm{Ks}},\mathrm{and }{\mathrm{I}}_{\mathrm{K}1}\right)$ from the start of the stimulus to the end of a beat $(\mathrm{t}=\mathrm{2,000 ms})$. The CaD90 is the calcium duration calculated from the peak of intracellular calcium concentration to its 90% value.

For one simulation, the variation of drug concentrations used was from zero to 4 times the maximum therapeutic concentration (cmax) for each drug (1 × increment). Therefore, combining two drugs could yield 25 drug concentration pairs. From 12 CiPA drugs, one might obtain $(12\times 12-12)/2=66$ unique drug combinations. In total, there are $66\times 25\times 100=1$ 65,000 simulations in this study. In addition, this study also includes an additional combined drug of hydroxychloroquine mixed with diltiazem for obtaining validation results of combined drugs’ effects, as reported by Choi et al.^37^. The combination of hydroxychloroquine and diltiazem was found to increase the QT prolongation, which was considered one important signature of TdP. The data of ${\mathrm{IC}}_{50}$ and $\mathrm{n}$ for hydroxychloroquine is obtained from the study of Delaunois et al.^29^, and the simulation protocol used is the same as the one used by 12 CiPA drugs. Additional simulations incorporating drug datasets similar to the one in the study of Delaunois et al.^29^ and Whittaker et al.^31^ (originated from the work of Crumb et al.^38^) are also performed as in the supplementary materials to enhance the comparative study of the drug combinations’ effects.

A simple classification procedure is used to assess the TdP risk of combined compounds using TdP metric score as the average value of qNets calculated from $1-4\times \mathrm{cmax}$ from single drug simulations of 12 CiPA drugs. The threshold values of qNet that categorize the combined drugs into low, intermediate, and high risk are obtained using the ordinal regression model proposed by Li et al.^39^. Furthermore, the features’ class from drug combination pairs is presented using drug combination maps. This study only shows the map of qNet as the main feature for TdP risk metric. The example of using drug combination maps for three drugs can be seen in Fig. 3 panel 1. The unique drug combinations are presented as blue boxes. The formula for the total number of unique drug combinations can be expressed as:

$${\text{unique combinations}} = \frac{{{\text{N}}\left( {{\text{N}} - 1} \right)}}{2}$$

where $\mathrm{N}$ is the number of drugs (in this case $\mathrm{N}=12$). The same-drug combinations (diagonal boxes in Fig. 3 panel 1) are not counted because they would represent only single-drug effects. Each predicted TdP group is plotted in separate combination maps, as shown in Fig. 3 panel 2.

## Results

**Figure 1:**
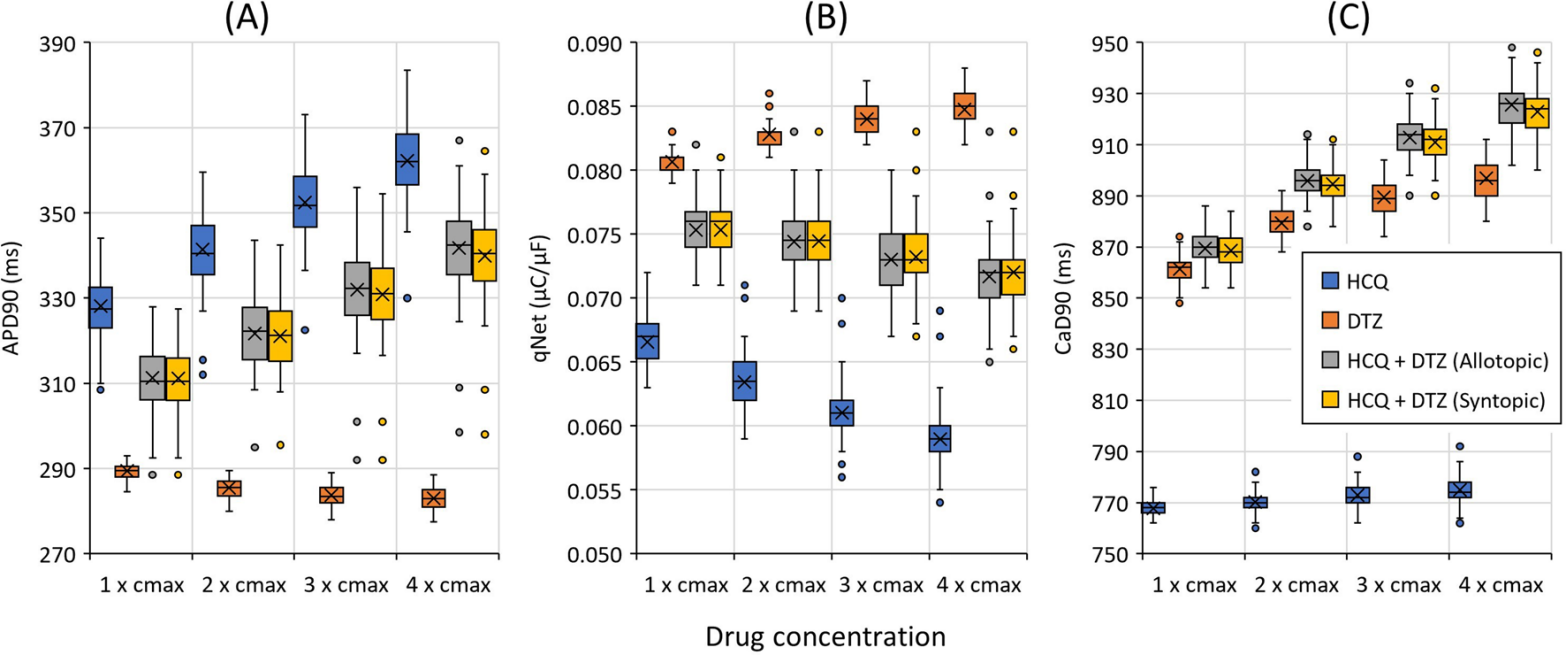
Features (APD90, qNet, and CaD90) as a function of drug concentrations for hydroxychloroquine, diltiazem, and combined hydroxychloroquine-diltiazem. Panel (**A**) shows the result of APD90, panel (**B**) for qNet, and panel (**C**) for CaD90. The labels in $\mathrm{x}$ axis represent the concentration of each compound incorporated in the simulation. For example, $3\times \mathrm{cmax}$ for the combination of compound A and B means that the concentration for each A and B is $3\times \mathrm{cmax}$. Furthermore, in a single box plot, the cross line refers to the average value of the feature; the lower, middle, and upper lines of the box refer to the first quartile, median, and third quartile, respectively; the upper and lower lines outside the box refer to maximum and minimum values excluding the outliers; circles represent the outliers.

The single and combined drug effects are compared by combining hydroxychloroquine and diltiazem, as shown by the box plot in Fig. 1 for APD90, qNet, and CaD90. For the results of APD90 in panel (A) of Fig. 1, hydroxychloroquine shows higher values of APD90 compared to diltiazem and their combinations. Furthermore, diltiazem yields the lowest APD90 values among them and displays the only negative trend of ADP90 values (APD shortening) as drug concentration increases. In $1\times \mathrm{cmax}$, the lowest APD90 value is 284.5 ms by diltiazem and the highest is 344 ms by hydroxychloroquine; and in $4\times \mathrm{cmax}$, it becomes 277.5 ms for diltiazem and 383.5 ms for hydroxychloroquine. The combined drugs (for both allotopic and syntopic models) show APD90 values in between with a positive trend (APD90 prolongation) as hydroxychloroquine, indicating combined drug’s effects on APD90 are more strongly influenced by hydroxychloroquine than diltiazem.

Furthermore, the qNet results are presented in panel (B) of Fig. 1. The diltiazem produces the highest qNet values, the hydroxychloroquine generates the lowest qNet data, and their combination yields anything in between. Interestingly, the only positive trend of qNet as a function of drug concentration is produced by diltiazem. In $1\times \mathrm{cmax}$, the highest qNet value is $0.082\mathrm{ \mu C}/\mathrm{\mu F}$ by diltiazem, and the lowest one is $0.063\mathrm{ \mu C}/\mathrm{\mu F}$ by hydroxychloroquine. In high concentration of $4\times \mathrm{cmax}$, the pattern is similar that the highest qNet is about $0.088\mathrm{ \mu C}/\mathrm{\mu F}$ by diltiazem, and the lowest is $0.055\mathrm{ \mu C}/\mathrm{\mu F}$. In addition, the combined hydroxychloroquine-diltiazem results in the qNet trend that follow the hydroxychloroquine’s qNet tendency towards higher concentration of drugs (negative direction), indicating that hydroxychloroquine affects more dominantly in lowering the qNet values of the combined drug than diltiazem.

**Figure 2:**
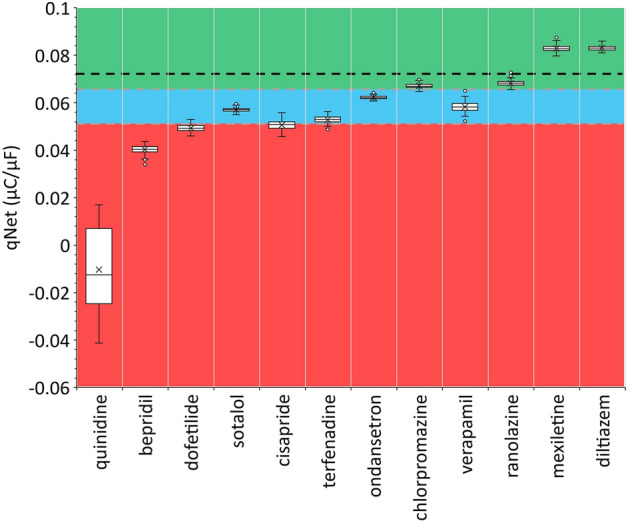
Distribution of qNet for 12 CiPA drugs (single drug effects). The qNet values shown in the figure were the average qNet values from drug concentration of $1-4\times \mathrm{cmax}$ as in Li et al.^39^. The upper horizontal grey dashed line represents the ${\mathrm{threshold}}_{1}=0.0652\mathrm{ \mu C}/\mathrm{\mu F}$ and the lower one for ${\mathrm{threshold}}_{2}=0.0516\mathrm{ \mu C}/\mathrm{\mu F}$. The black dashed line depicts the control (drug free) value of qNet at $0.072\mathrm{ \mu C}/\mathrm{\mu F}$. qNet values higher than ${\mathrm{threshold}}_{1}$ are classified as low risk, between ${\mathrm{threshold}}_{1}$ and ${\mathrm{threshold}}_{2}$ are classified as intermediate risk, and below ${\mathrm{threshold}}_{2}$ for high-risk drugs.

Moreover, we can observe from panel (C) of Fig. 1 that the combined drugs can yield a higher value of CaD90 compared to results from single drug effects, while the least CaD90 values were produced by hydroxychloroquine. In $1\times \mathrm{cmax}$, the CaD90 values ranged from 762 ms (yielded by hydroxychloroquine) to 886 ms (generated by combined hydroxychloroquine and diltiazem) from the allotopic model and 884 ms from the syntopic model. The CaD90 values by hydroxychloroquine for higher drug concentrations show a small increment with a maximum of 786 at $4\times \mathrm{cmax}$. On the other hand, diltiazem and combined hydroxychloroquine-diltiazem show quite a substantial rise in CaD90 values as the drug concentration increases. In addition, the data of CaD90 from hydroxychloroquine show a considerable gap to both diltiazem and combined one, indicating combined drug’s effects are affected more by diltiazem than hydroxychloroquine in producing high CaD90 values. Finally, datasets from some previous studies of Delaunois et al.^29^ and Whittaker et al.^31^ are simulated as in supplementary materials Figure S2 and Figure S3 and show consistent results in producing APD90, CaD90, and qNet values. The results of averaged qNet distribution for 12 CiPA drugs are shown in Fig. 2. The high-risk drugs (quinidine, bepridil, dofetilide, and sotalol) generally generate low qNet values, except the sotalol that produces qNet between ${\mathrm{threshold}}_{1}$ and ${\mathrm{threshold}}_{2}$. Furthermore, the intermediate-risk drugs (cisapride, terfenadine, ondansetron, and chlorpromazine) are typically within two thresholds with some exceptions for cisapride, terfenadine, and ranolazine that generate some qNet data outside this region. Finally, the low-risk drugs (ranolazine, mexiletine, diltiazem) produce qNet values mostly above ${\mathrm{threshold}}_{1}$ except verapamil that yields qNet within the two thresholds. The two threshold values are utilized for further analysis in drug combination maps of combined drugs’ effects.

**Figure 3:**
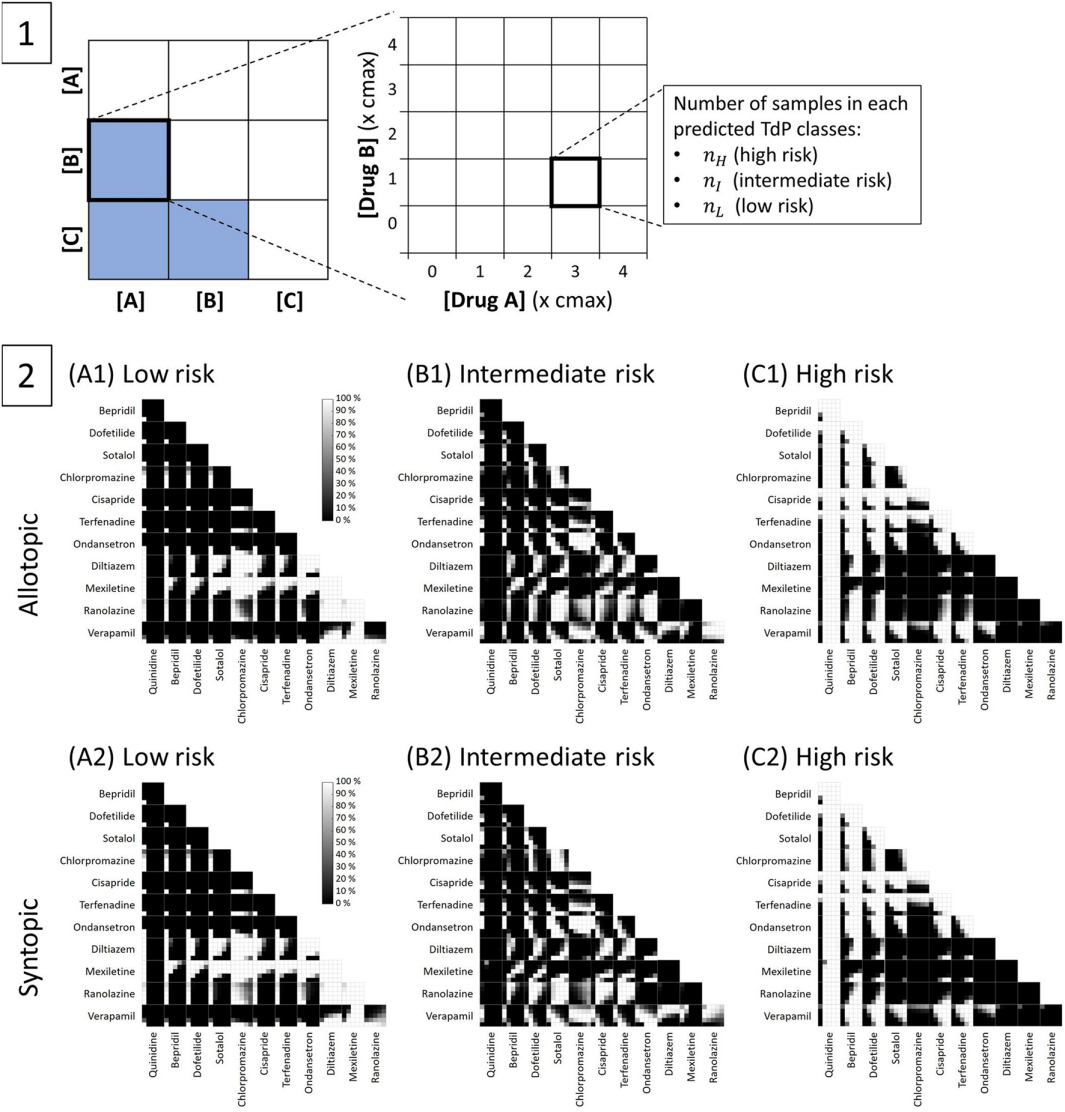
The maps of TdP risk prediction. Panel 1 show the an example of detailed description of the combination maps. There are three unique combinations (blue boxes) that each contains various drug concentration pairs ranging from $0-4\times \mathrm{cmax}$. Each blue box represents a map with a vertical and horizontal axis of $0-4\times \mathrm{cmax}$ drug concentration variations. The axis value of $0\times \mathrm{cmax}$ indicates the drug-free simulations that resemble single drug effects. For example, if the concentration for drug A is $0\times \mathrm{cmax}$, the combined effect of mixed-drug A and B will result in a single effect of drug B. Furthermore, in each drug concentration pair, the total number of samples is 100 that is splitted into three different predicted TdP classes (${n}_{H}$ for samples predicted as high risk compounds, ${n}_{I}$ as intermediate risk, and ${n}_{L}$ as low risk). Each group of predicted TdP risk can be plotted into separate combination maps as in Panel 2. Panel 2 show the maps for TdP prediction of allotopic and syntopic models. Three panels (A, B, and C) for each model show different classes of predicted TdP risk based on the value of qNet on each pairwise drug sample. Panel A1 and A2 on the left show the maps of resulted compounds predicted as low risk, panel B1 and B2 for compound predicted as intermediate risk, and panel C1 and C2 for compounds predicted as high risk. Each panel (A or B or C) of predicted TdP class shows a unique combination of 12 CiPA drugs, resulting in 66 drug combination maps containing 25 drug concentration pairs. Each combination of drug concentrations contains 100 samples of data of qNets from which one can classify whether it is low, intermediate, or high-risk compounds using threshold values of ${\mathrm{threshold}}_{1}$ and ${\mathrm{threshold}}_{2}$. The map's color represents the percentage of samples categorized as low, intermediate, or high-risk compounds. Black color (0%) shows no sample falls under the corresponding category; white color (100%) represents all samples classified as related risk category. The consistency of the results can be assessed from the single drug effects (drug concentration is $0\times \mathrm{cmax}$) in the first axis of each combination map.

The maps of TdP risk prediction by allotopic and syntopic models on 66 unique drug combinations can be seen in Fig. 3. The combinations of both high-risk drugs mostly yield high-risk compounds, as shown in the upper part of all panels (for both allotopic and syntopic models). However, a mixture of sotalol $\left(1\times \mathrm{cmax}\right)$ with dofetilide $\left(1\times \mathrm{cmax}\right)$ can produce some intermediate-risk compounds. Furthermore, the blends of high and intermediate-risk drugs also primarily generate high-risk and some intermediate-risk compounds. When combining chlorpromazine and ondansetron with dofetilide and sotalol, they can produce intermediate-risk compounds when the high-risk components (dofetilide or sotalol) are at low concentrations. Moreover, combinations of high and low-risk drugs can yield all categories of compounds (low, intermediate, and high) with various combinations of drug concentrations. 100% low-risk regions can be yielded when combining diltiazem $\left(1-4\times \mathrm{cmax}\right)$ with dofetilide $\left(1\times \mathrm{cmax}\right)$ and sotalol $\left(1-2\times \mathrm{cmax}\right)$, mexiletine $\left(3-4\times \mathrm{cmax}\right)$ with bepridil $\left(1\times \mathrm{cmax}\right)$, mexiletine $\left(2-4\times \mathrm{cmax}\right)$ with dofetilide $\left(1-2\times \mathrm{cmax}\right)$, and mexiletine $\left(1-4\times \mathrm{cmax}\right)$ with sotalol $\left(1-4\times \mathrm{cmax}\right)$. However, the 100% intermediate regions are produced partially by all combinations of high and low-risk drugs. Finally, the 100% high-risk regions are yielded by the mixture of quinidine $\left(1-4\times \mathrm{cmax}\right)$ with all low-risk drugs, bepridil $\left(1-4\times \mathrm{cmax}\right)$ with verapamil $\left(1-4\times \mathrm{cmax}\right)$, bepridil $\left(2-4\times \mathrm{cmax}\right)$ with ranolazine $\left(1-4\times \mathrm{cmax}\right)$, bepridil $\left(3-4\times \mathrm{cmax}\right)$ with mexiletine $\left(1\times \mathrm{cmax}\right)$ and diltiazem $\left(1-4\times \mathrm{cmax}\right)$, dofetilide $\left(1-4\times \mathrm{cmax}\right)$ with verapamil $\left(1-4\times \mathrm{cmax}\right)$, dofetilide $\left(3-4\times \mathrm{cmax}\right)$ with ranolazine $\left(1-4\times \mathrm{cmax}\right)$, and dofetilide $\left(4\times \mathrm{cmax}\right)$ with mexiletine $\left(1\times \mathrm{cmax}\right)$.

The combinations of both intermediate-risk drugs mainly generate high and intermediate-risk compounds. Combining chlorpromazine with ondansetron can produce a 100% intermediate-risk region, as shown in panel (B). Furthermore, the mixture of cisapride and terfenadine shows 100% high-risk regions, while other combinations mostly yield both high and intermediate-risk regions in various drug concentration pairs. Moreover, the mixtures of intermediate and low-risk drugs can generate all types of compounds’ risks (low, intermediate, and high) under various drug concentrations. The combinations with the most 100% low-risk regions generated are chlorpromazine $\left(1-4\times \mathrm{cmax}\right)$ with diltiazem $\left(1-4\times \mathrm{cmax}\right)$ and mexiletine $\left(1-4\times \mathrm{cmax}\right)$, mexiletine $\left(2-4\times \mathrm{cmax}\right)$ with cisapride $\left(1-4\times \mathrm{cmax}\right)$ and terfenadine $\left(1-4\times \mathrm{cmax}\right)$, ondansetron $\left(1-4\times \mathrm{cmax}\right)$ with mexiletine $\left(1-4\times \mathrm{cmax}\right)$ and diltiazem $\left(1-4\times \mathrm{cmax}\right)$. In addition, 100% intermediate-risk regions are generated following various drug concentration pairs of intermediate-low-risk drug combinations. Finally, 100% high-risk regions produced mainly by the teams of verapamil $\left(1-4\times \mathrm{cmax}\right)$ with cisapride $\left(1-4\times \mathrm{cmax}\right)$ and terfenadine $\left(1-4\times \mathrm{cmax}\right)$, and verapamil $\left(2-4\times \mathrm{cmax}\right)$ with ondansetron $\left(2-4\times \mathrm{cmax}\right)$. The last combination pairs are both low-risk drugs that generate mostly low-risk with some possibility for intermediate-risk compounds. The 100% low-risk regions are produced by combining diltiazem $\left(1-4\times \mathrm{cmax}\right)$ with verapamil $\left(1-2\times \mathrm{cmax}\right)$, ranolazine $\left(1-4\times \mathrm{cmax}\right)$, and mexiletine $\left(1-4\times \mathrm{cmax}\right)$, mexiletine $\left(1-4\times \mathrm{cmax}\right)$ with verapamil $\left(1-4\times \mathrm{cmax}\right)$ and ranolazine $\left(1-4\times \mathrm{cmax}\right)$. The combination of verapamil $\left(2-3\times \mathrm{cmax}\right)$ with ranolazine $\left(1-4\times \mathrm{cmax}\right)$ mostly generates intermediate-risk compounds.

**Figure 4:**
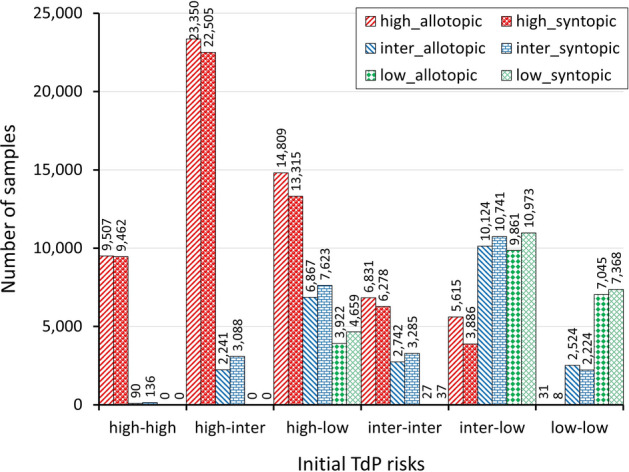
Bar chart of TdP risks of compounds from the combination of high, intermediate, and low-risk drugs. The horizontal axis represents the pairs of initial TdP risks of combined drugs, the vertical axis is the number of samples or compounds resulting from drug combinations, and the colored bars represent the predicted risk of compounds resulting from a combination of initial TdP risks of drugs. The value above each bar indicates number of samples of the corresponding bar.

Finally, Fig. 4 presents the summary results of drug-risk combinations. It is shown that combinations of high-high and high-intermediate do not produce low-risk compounds. In addition, some intermediate-risk compounds are produced (90 samples from allotopic and 136 samples from syntopic) when combining both high-risk drugs that mainly occur at the mixture of dofetilide $\left(1\times \mathrm{cmax}\right)$ and sotalol $\left(1\times \mathrm{cmax}\right)$. Also, the pair of both low-risk drugs may result in high-risk compounds (31 samples predicted by the allotopic model and 8 samples predicted by the syntopic model) that typically occur at the combination of ranolazine $\left(1-2\times \mathrm{cmax}\right)$ with verapamil $\left(4\times \mathrm{cmax}\right)$ from allotopic model and diltiazem $\left(1\times \mathrm{cmax}\right)$ with verapamil $\left(4\times \mathrm{cmax}\right)$ from the syntopic model. Moreover, when combining both intermediate-risk drugs, one may produce low-risk compounds (27 samples predicted by the allotopic model and 37 samples predicted by the syntopic model), especially from a combination of chlorpromazine $\left(1\times \mathrm{cmax}\right)$ with ondansetron $\left(1\times \mathrm{cmax}\right)$. Finally, various risk (low, intermediate, and high) compounds can be found when combining high-low and intermediate-low risk drugs.

## Discussion

The qNet values from Fig. 2 indicate that the low qNet is more associated with high-risk drugs, and the high qNet is more related to low-risk drugs. Furthermore, from the results of features represented in panel (B) of Fig. 1, we can observe that the mixture of hydroxychloroquine-diltiazem tends to decrease the value of qNet as drug concentration increases which means it is strongly associated with high-risk drugs. It agrees with the findings of the study of Choi et al.^37^ that showed hydroxychloroquine and diltiazem interact in the tendency to increase QT prolongation, which is well-known to be the significant influence of high-risk drugs. In addition, a study from Montnach et al.^32^ showed that a combination of azithromycin and hydroxychloroquine could generate longer QT duration compared to single drug effects, that is consistent with our results shown in Figure S3 in supplementary materials. However, the combined drugs affect biomarker features differently, as shown in Fig. 1 that hydroxychloroquine-diltiazem shows a similar trend to hydroxychloroquine for APD90 and qNet. In contrast, CaD90 is affected more by diltiazem. Since the drug combination’s effect is applied to multiple ion channels, the overall effect can be very nonlinear and may result in various responses for each biomarker. For example, qNet, as a well-known TdP metric score, can be affected strongly by the change of ${\mathrm{K}}_{\mathrm{r}}$ channel^6^, and likewise, the variation of CaD90 is strongly influenced by the alteration of $\mathrm{CaL}$ channel^40^. Therefore, one may interpret that from panel (B) in Fig. 1, the hydroxychloroquine affects more dominantly when combined with diltiazem in the Kr channel. From panel (C), oppositely, the diltiazem influences more strongly when combined with hydroxychloroquine in the $\mathrm{CaL}$ channel.

Furthermore, drug combinations shown in Fig. 3 and the summary results in Fig. 4 show that combining a variety of TdP-risk drugs can produce drug compounds with different TdP risk categories compared to their original classes of TdP risk. Various TdP-risk compounds can be made mainly by combining the well-known high with low-risk drugs or intermediate with low-risk drugs. This finding may be advantageous, especially when the specific drug is hard to find in some regions. Also, when a new, infectious TdP-related disease spreads, combining the existing drugs to fight the disease may provide a suitable alternative instead of finding new compounds for targeting specific illnesses that may take more time to research, test, and produce.

Moreover, the in silico assessment of risks of combined drugs presented in this work may not be limited to TdP risk only. The effects of other types of combined medicines may target different kinds of organs or cells. Therefore, the in silico risk or efficacy assessment of combined drugs may be used for other diseases as well, starting by gathering dose–response data of the effects of single drugs and using the $I{C}_{50}$ and Hill coefficient $n$ obtained from the fitting and bootstrapping to assess effects of combined drugs using simulation. The recent emergence of the COVID-19 pandemic has been an excellent example of the use of combined drugs during therapies. For instance, Chakraborty et al.^41^ reported that combining drugs incorporating remdesivir, tocilizumab, dexamethasone, and baricitinib might result in beneficial outcomes for COVID-19 patients. In addition, the study by Kalil et al.^42^ reported that combined treatment of baricitinib with remdisivir to COVID-19 patients undergoing in-hospital medication shows better results when compared to the single drug of remdesivir. Another study from Benfield et al.^43^ also reported that the combined remdesivir and dexamethasone for COVID-19 patients show a reduction in 30-day mortality. In addition, combined medications can also provide a good alternative for existing, well-known diseases. For example, in cases for treating cardiovascular disease (CVD), a report from Yusuf et al.^44^ showed that a combination of drug therapy in addition to proper lifestyle man results in a considerable amount of reduction of CVD by 70–80%. The study found that combined medication incorporating aspirin and statin shows substantial beneficial results for treating CVD by reducing risk factors and enhancing medication adherence in preventing CVD. With various uses of combined medications for numerous diseases, in silico assessment using combined drug model can provide a better understanding of the behavior of compounds as the product of combining existing drugs.

Moreover, Joseph et al.^45^ reported that fixed-dose combination therapy approaches significantly lower CVD in populations without vascular disease. The blood pressure-lowering drugs, statin, and aspirin formulations show the most significant decreases in the risk of CVD. Our proposed in silico assessment may become an excellent approach to designing the fixed-dose combination therapy using the drug combination maps as in Fig. 3. For illustration, by taking a straight line from the origin of the drug combination map, one can have a fixed drug concentration ratio. The effect of drug combination from the chosen ratio can be assessed to model the therapy strategy necessary for patients.

The limitations of this study and associated future works are as follows. First, the most important thing to notice is that because of the limited experimental data for assessing combined drugs, this study's results may not vigorously represent the actual risk of drug combinations. One may need experimental data on true TdP level of combined drugs to accurately validate the mathematical model of drug combination and predict the risk of other combined compounds before using it for clinical practice. Moreover, this study assumed a fixed cmax value of the drugs in simulation, although the cmax of drugs may depend on different PK conditions and tissue localization. A more precise TdP prediction of polypharmacy should be patient-specific to set the cmax value accurately. Furthermore, this study only considered qNet as a primary TdP metric obtained from electrophysiological simulation of single drug effects. Adding other biomarker features in analysis with a more advanced machine learning method may reveal a more precise prediction of TdP risk of combined drugs. This study also did not consider hERG dynamic model^7^ in TdP risk assessment. Combining the dynamic hERG binding parameters of two drugs (by incorporating one gating model with two different binding models) could also allow TdP risk prediction that requires only single drug information. Furthermore, the authors did not assess the mechanosensitive ion channels that might alter the overall electrophysiological responses of the cardiac cells. Some examples of the corresponding channels are mechanosensitive potassium channel^46^ and stretch-activated ion channels^47^. Therefore, one may need to conduct an electromechanical simulation to evaluate the effects of the combined drugs when considering the mechanosensitive ion channels. Lastly, when the experimental (dose–response) drug combination data for several ion channels is sufficiently available, one may incorporate a more advanced DDIs model such as GPDI^25^ to assess TdP risk of combined drugs more realistically.

## Supplementary Information

Supplementary Information 1.

Supplementary Information 2.

## Data Availability

The original contributions presented in the study are included in the article/supplementary material; further inquiries can be directed to the corresponding author/s.
