# Supplementary material for: In silico assessment on TdP risks of drug combinations under CiPA paradigm
Source: Sci Rep. 2023 Feb 20;13:2924. doi: 10.1038/s41598-023-29208-5 (PMC9940090; doi:10.1038/s41598-023-29208-5)
Supplement: Supplementary file 2 — Supplementary Information 2. [file 41598_2023_29208_MOESM2_ESM.docx]

***In Silico* Assessment on TdP Risks of Drug Combinations under CiPA Paradigm**

**Ali Ikhsanul Qauli^1,2^, Aroli Marcellinus^2^, Muhammad Aldo Setiawan^1^, Andi Faiz Naufal Zain^1^, Azka Muhammad Pinandito^1^, and Ki Moo Lim^2,3,4*^**

^1^Universitas Airlangga, Faculty of Advanced Technology and Multidiscipline, Department of Robotics and Artificial Intelligence Engineering, Surabaya, Indonesia

^2^Kumoh National Institute of Technology, IT convergence engineering, Gumi, 39253, Republic of Korea.

^3^Kumoh National Institute of Technology, Medical IT convergence engineering, Gumi, 39253, Republic of Korea.
^4^Meta Heart Inc., Gumi, 39253, Republic of Korea.

ali.ikhsanul.q@ftmm.unair.ac.id

armarcell@kumoh.ac.kr

m.aldo.setiawan@ftmm.unair.ac.id

andi.faiz.naufal-2020@ftmm.unair.ac.id

azka.muhammad.pinandito-2020@ftmm.unair.ac.id

*kmlim@kumoh.ac.kr

**Supplementary materials**

Expression of ion channel currents ^1–3^

The fast sodium current $\left( I_{Na} \right)$:

$$I_{Na}=G_{Na}\left( V_{m}-E_{Na} \right)m^{3}\left( \left( 1-\phi_{INa,CaMK} \right)hj+\phi_{INa,CaMK}h_{CaMK}j_{CaMK} \right)$$

The late sodium current $\left( I_{NaL} \right)$:

$$I_{NaL}=G_{NaL}\left( V_{m}-E_{Na} \right)m_{L}\left( \left( 1-\phi_{INaL,CaMK} \right)h_{L}+\phi_{INaL,CaMK}h_{L,CaMK} \right)$$

Transient outward potassium current $\left( I_{to} \right)$:

$$I_{to}=G_{to}\left( V_{m}-E_{K} \right)\left( \left( 1-\phi_{Ito,CaMK} \right)ai+\phi_{Ito,CaMK}a_{CaMK}i_{CaMK} \right)$$

L-type calcium current $\left( I_{CaL} \right)$:

$$I_{CaL}=P_{Ca}z_{Ca}^{2}\frac{V_{m}F^{2}}{RT}\frac{\gamma_{Cai}\left[ Ca^{2+} \right]_{ss}\times\exp\left( \frac{z_{Ca}V_{m}F}{RT} \right)-\gamma_{Cao}\left[ C^{2+} \right]_{o}}{e^{\frac{z_{Ca}V_{m}F}{RT}}-1}d\left( 1-\phi_{ICaL,CaMK} \right)\left( f\left( 1-n \right)+f_{Ca}nj_{Ca} \right)+P_{Ca,CaMK}z_{Ca}^{2}\frac{V_{m}F^{2}}{RT}\frac{\gamma_{Cai}\left[ Ca^{2+} \right]_{ss}\times\exp\left( \frac{z_{Ca}V_{m}F}{RT} \right)-\gamma_{Cao}\left[ C^{2+} \right]_{o}}{e^{\frac{z_{Ca}V_{m}F}{RT}}-1}d\phi_{ICaL,CaMK}\left( f_{CaMK}\left( 1-n \right)+f_{Ca,CaMK}nj_{Ca} \right)$$

Sodium current through L-type calcium channel $\left( I_{CaNa} \right)$:

$$I_{CaNa}=P_{CaNa}z_{Na}^{2}\frac{V_{m}F^{2}}{RT}\frac{\gamma_{Nai}\left[ Na^{+} \right]_{ss}\exp\left( \frac{z_{Na}V_{m}F}{RT} \right)-\gamma_{Nao}\left[ Na^{+} \right]_{o}}{e^{\frac{z_{Na}V_{m}F}{RT}}-1}d\left( 1-\phi_{ICaL,CaMK} \right)\left( f\left( 1-n \right)+f_{Ca}nj_{Ca} \right)+P_{CaNa,CaMK}z_{Na}^{2}\frac{V_{m}F^{2}}{RT}\frac{\gamma_{Nai}\left[ Na^{+} \right]_{ss}\exp\left( \frac{z_{Na}V_{m}F}{RT} \right)-\gamma_{Nao}\left[ Na^{+} \right]_{o}}{e^{\frac{z_{Na}V_{m}F}{RT}}-1}d\phi_{ICaL,CaMK}\left( f_{CaMK}\left( 1-n \right)+f_{Ca,CaMK}nj_{Ca} \right)$$

Potassium current through L-type calcium channel $\left( I_{CaK} \right)$:

$$I_{CaK}=P_{CaK}z_{K}^{2}\frac{V_{m}F^{2}}{RT}\frac{\gamma_{Ki}\left[ K^{+} \right]_{ss}\exp\left( \frac{z_{K}V_{m}F}{RT} \right)-\gamma_{Ko}\left[ K^{+} \right]_{o}}{e^{\frac{z_{K}V_{m}F}{RT}}-1}d\left( 1-\phi_{ICaL,CaMK} \right)\left( f\left( 1-n \right)+f_{Ca}nj_{Ca} \right)+P_{CaK,CaMK}z_{K}^{2}\frac{V_{m}F^{2}}{RT}\frac{\gamma_{Ki}\left[ K^{+} \right]_{ss}\exp\left( \frac{z_{K}V_{m}F}{RT} \right)-\gamma_{Ko}\left[ K^{+} \right]_{o}}{e^{\frac{z_{K}V_{m}F}{RT}}-1}d\phi_{ICaL,CaMK}\left( f_{CaMK}\left( 1-n \right)+f_{Ca,CaMK}nj_{Ca} \right)$$

Rapid delayed rectifier current $\left( I_{Kr} \right)$:

$$I_{Kr}=G_{Kr}\sqrt{\frac{\left[ K^{+} \right]_{o}}{5.4}}x_{r}R_{Kr}(V_{m}-E_{K})$$

Slow-delayed rectifier current $\left( I_{Ks} \right)$:

$$I_{Ks}=G_{Ks}\left( 1+\frac{0.6}{1+\left( \frac{3.8\times{10}^{-5}}{\left[ Ca^{2+} \right]_{i}} \right)^{1.4}} \right)x_{s1}x_{s2}(V_{m}-E_{Ks})$$

Inward rectifier potassium current $\left( I_{K1} \right)$:

$$I_{K1}=G_{K1}\sqrt{\left[ K^{+} \right]_{o}}x_{K1}R_{K1}(V_{m}-E_{K})$$

Sodium-calcium exchange current $\left( I_{NaCa} \right)$:

$$I_{NaCa}=G_{NaCa}\times0.8\times allo_{i}\times\left( z_{Na}J_{NaCa,Na,i}+z_{Ca}J_{NaCa,Ca,i} \right)+G_{NaCa}\times0.2\times allo_{ss}\times\left( z_{Na}J_{NaCa,Na,ss}+z_{Ca}J_{NaCa,Ca,ss} \right)$$

Sodium ATPase current $\left( I_{NaK} \right)$:

$$I_{NaK}=30\left( z_{Na}J_{NaK,Na}+z_{K}J_{NaK,K} \right)$$

Background currents $\left( I_{Nab} \right)$,$\left( I_{Cab} \right)$, and $\left( I_{Kb} \right)$ :

$$I_{Nab}=P_{Nab}z_{Na}^{2}\frac{V_{m}F^{2}}{RT}\frac{\left[ Na^{+} \right]_{i}\exp\left( \frac{z_{Na}V_{m}F}{RT} \right)-\left[ Na^{+} \right]_{o}}{e^{\frac{z_{Na}V_{m}F}{RT}}-1}$$

$$I_{Cab}=P_{Cab}z_{Ca}^{2}\frac{V_{m}F^{2}}{RT}\frac{\gamma_{Cai}\left[ Ca^{2+} \right]_{i}\times\exp\left( \frac{z_{Ca}V_{m}F}{RT} \right)-\gamma_{Cao}\times\left[ C^{2+} \right]_{o}}{e^{\frac{z_{Ca}V_{m}F}{RT}}-1}$$

$$I_{Kb}=G_{Kb}x_{Kb}\left( V_{m}-E_{K} \right)$$

Sarcolemma calcium pump current $\left( I_{pCa} \right)$:

$$I_{pCa}=G_{pCa}\frac{\left[ Ca^{2+} \right]_{i}}{0.0005+\left[ Ca^{2+} \right]_{i}}$$

A more detailed explanation of the formulas and constants of the cardiac cell model can be found in the supplementary materials section from O’Hara et al.^1^.

In this study, we utilized the scaling factors from Dutta et al.^3^ and the cmax values for the corresponding drugs, as shown in **Table S1** and **Table S2**.

Table S1 Comparison of scaling between cardiac cell models and original model by O’Hara et al.

| **Scaled maximum conductance** | **Model by Li et al.**^2^ | **Model by Dutta et al.**^3^ |
| --- | --- | --- |
| $G_{Kr}$ | 0.9 | 1.013 |
| $G_{Ks}$ | 1.0 | 1.870 |
| $G_{K1}$ | 1.0 | 1.698 |
| $P_{Ca}$ | 1.0 | 1.007 |
| $G_{NaL}$ | 1.0 | 2.661 |

Table S2 The cmax values and risks of 12 CiPA drugs

| **Drug** | **TdP risk category**^2^ | **cmax (nM)**^4^ |
| --- | --- | --- |
| Quinidine | High | 3237 |
| Bepridil | High | 33 |
| Dofetilide | High | 2 |
| Sotalol | High | 14690 |
| Chlorpromazine | Intermediate | 38 |
| Cisapride | Intermediate | 2.6 |
| Terfenadine | Intermediate | 4 |
| Ondansetron | Intermediate | 139 |
| Diltiazem | Low | 122 |
| Mexiletine | Low | 4129 |
| Ranolazine | Low | 1948.2 |
| Verapamil | Low | 81 |

For the comparison to previous studies, we utilized the following dataset in simulations:

Table S3 Drugs in dataset 1

| **Drug** | **cmax (nM)** |
| --- | --- |
| Hydroxychloroquine^5^ | 495^6^ |
| Chloroquine^5^ | 410^7^ |
| Azithromycin^5^ | 760^8,9^ |
| Erythromycin^5^ | 3460^9,10^ |

Note that the drug dataset 1 in **Table S3** is from dataset #1 from Delaunois et al.^5^ paper.

Table S4 Drugs in dataset 2

| **Drug** | **cmax (nM)** |
| --- | --- |
| Hydroxychloroquine^5^ | 495^6^ |
| Azithromycin^11^ | 1,937^11^ |
| Chloroquine^11^ | 660^11^ |
| Lopinavir^11^ | 704^11^ |
| Moxifloxacin^11^ | 4,111^11^ |
| Ritonavir^11^ | 437^11^ |
| Quinine^11^ | 3,956.7^11^ |

The first comparison results between experimental data and DDIs models (allotopic and syntopic model) can be seen in **Figure S1**.


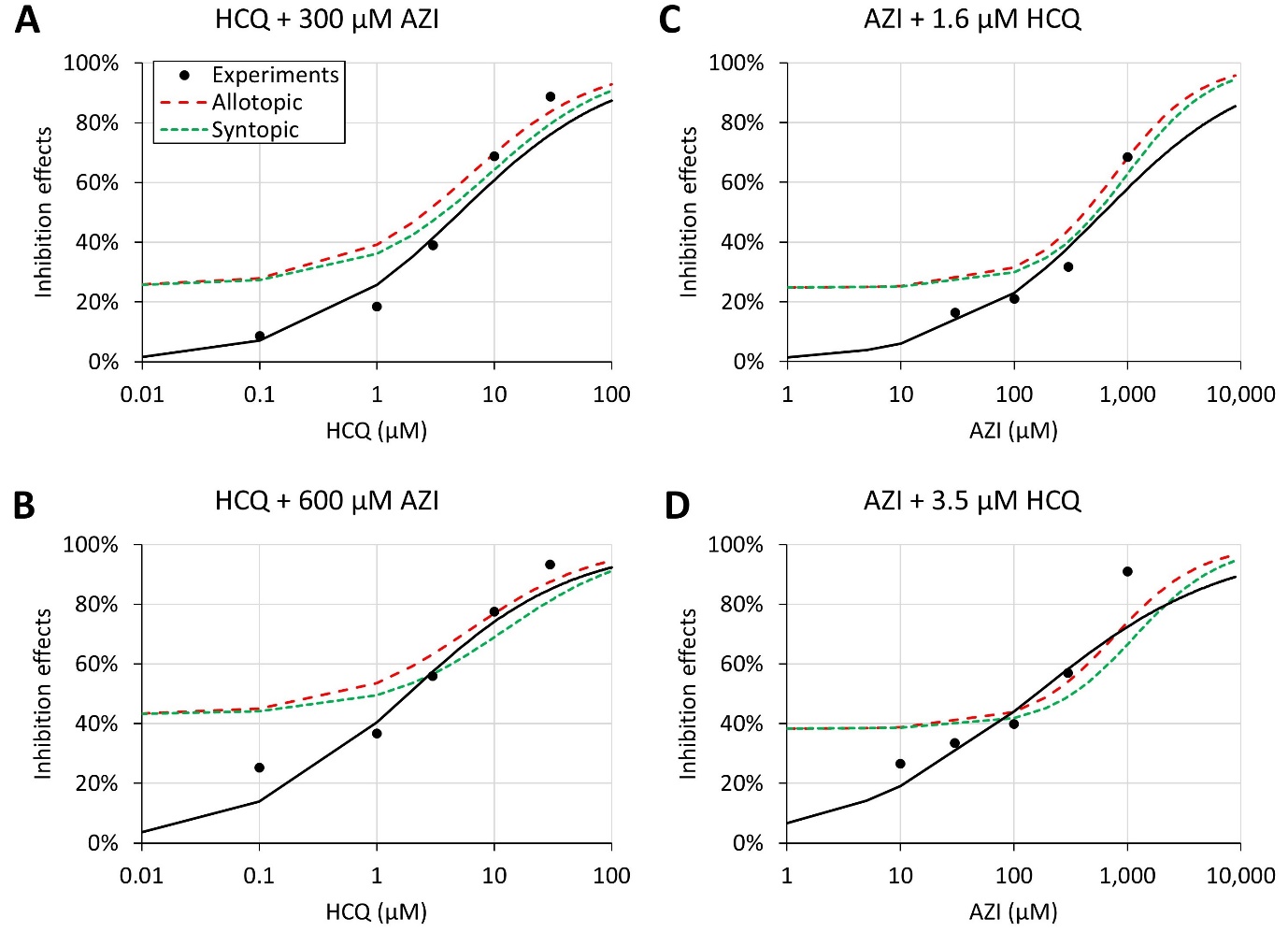


Figure S1 Comparison results of predictions from allotopic and syntopic model of drug-drug interactions with data from experiment on hERG channel (Figure 1 of paper from Delaunois et al.^5^). The drugs incorporated in the simulation were hydroxychloroquine (HCQ) and azithromycin (AZI). The experimental data shown in the figure is the mean value of the inhibition effect as a function of drug concentrations. The black line represents fitted line of the data generated from mean value and its standard error of mean.

Results from **Figure S1** shows that the drug combination models (allotopic and syntopic) generate quite accurate inhibition effects relative to fitted line from experimental data especially on high drug concentration. However, there are overestimated values of blocking effects of the model compared to experimental results especially in low drug concentration that is part of the model’s limitations.


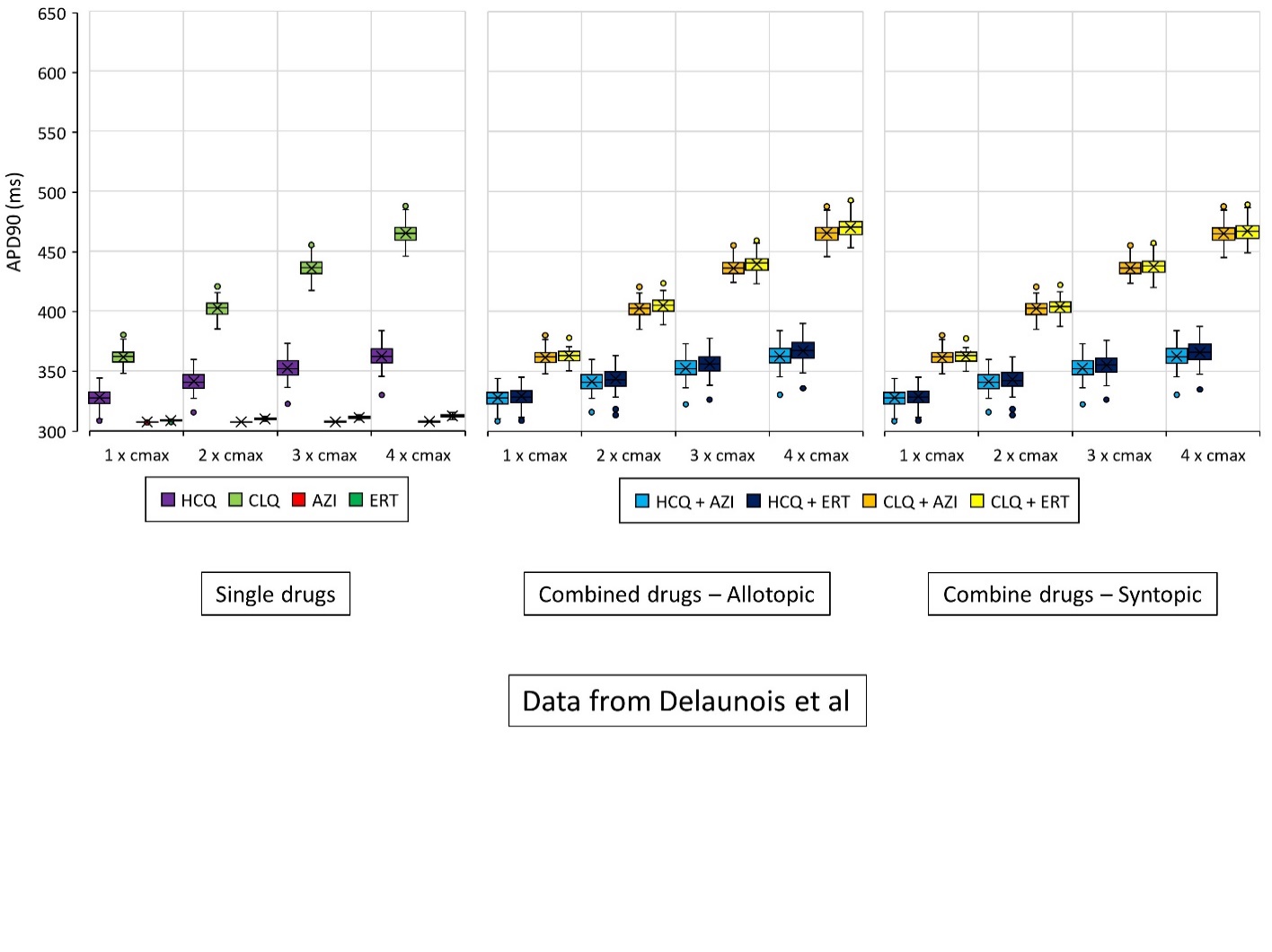


**Combine drugs – Syntopic**

**Combine drugs – Allotopic**

**Single drugs**


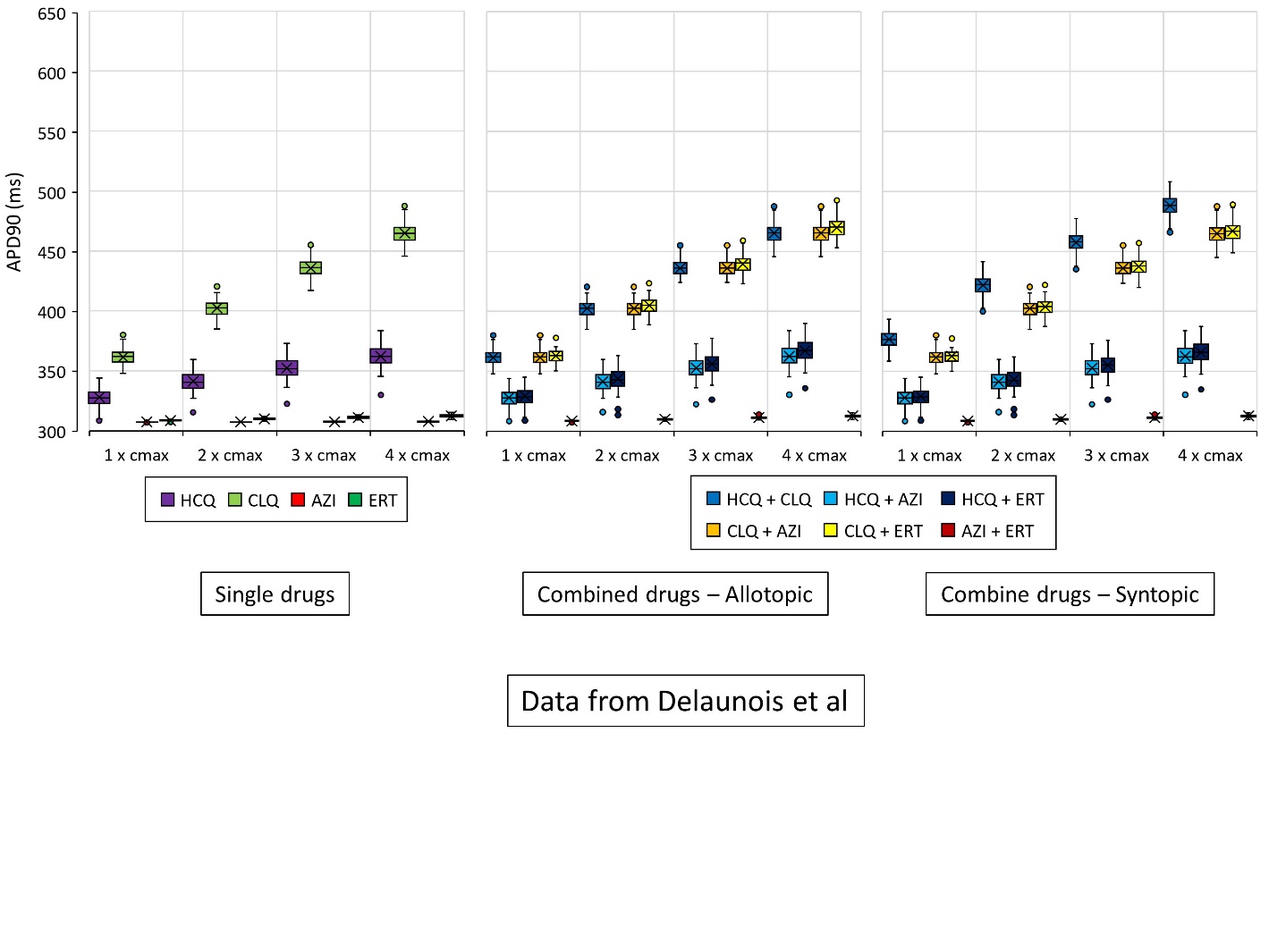

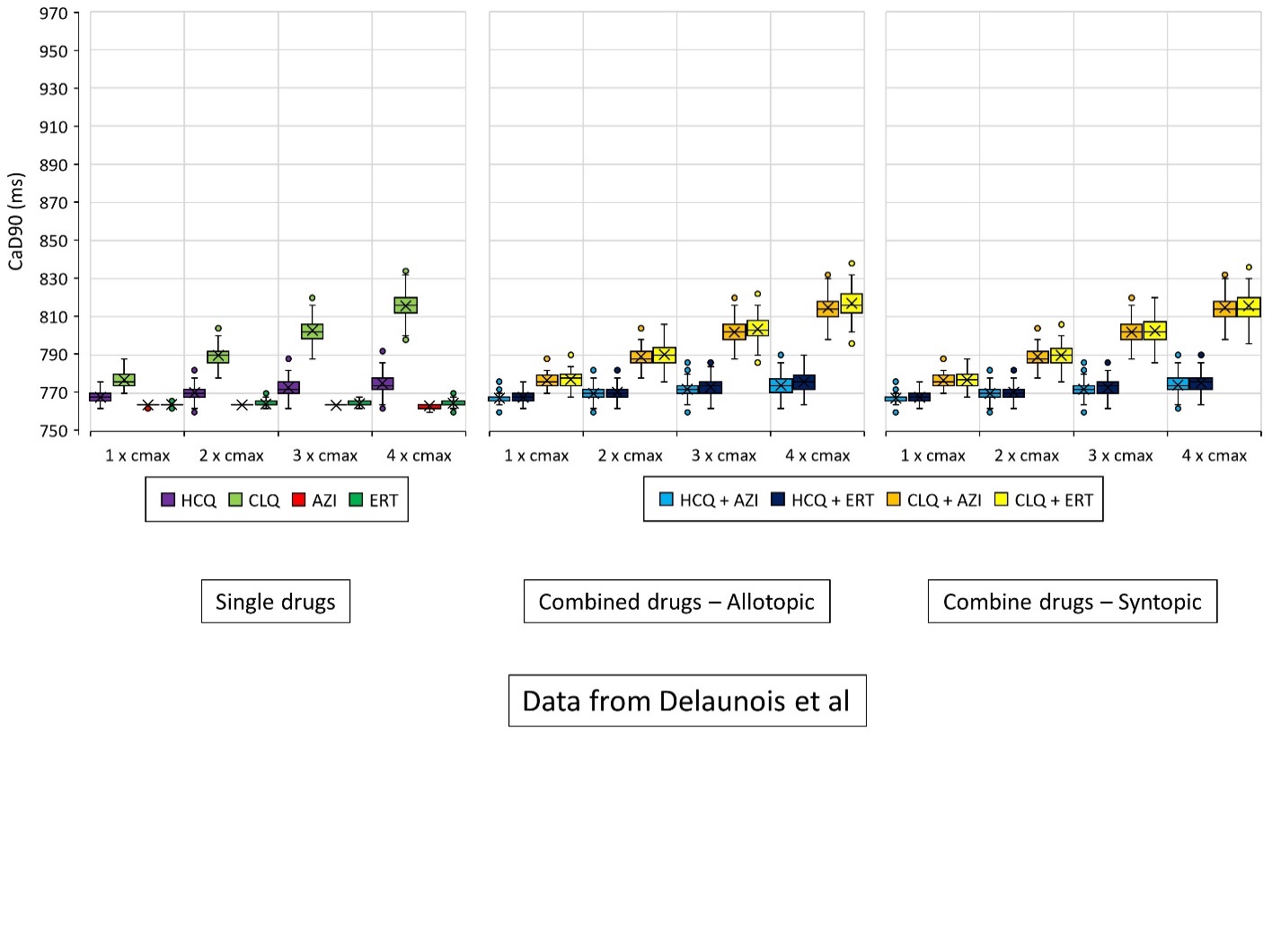

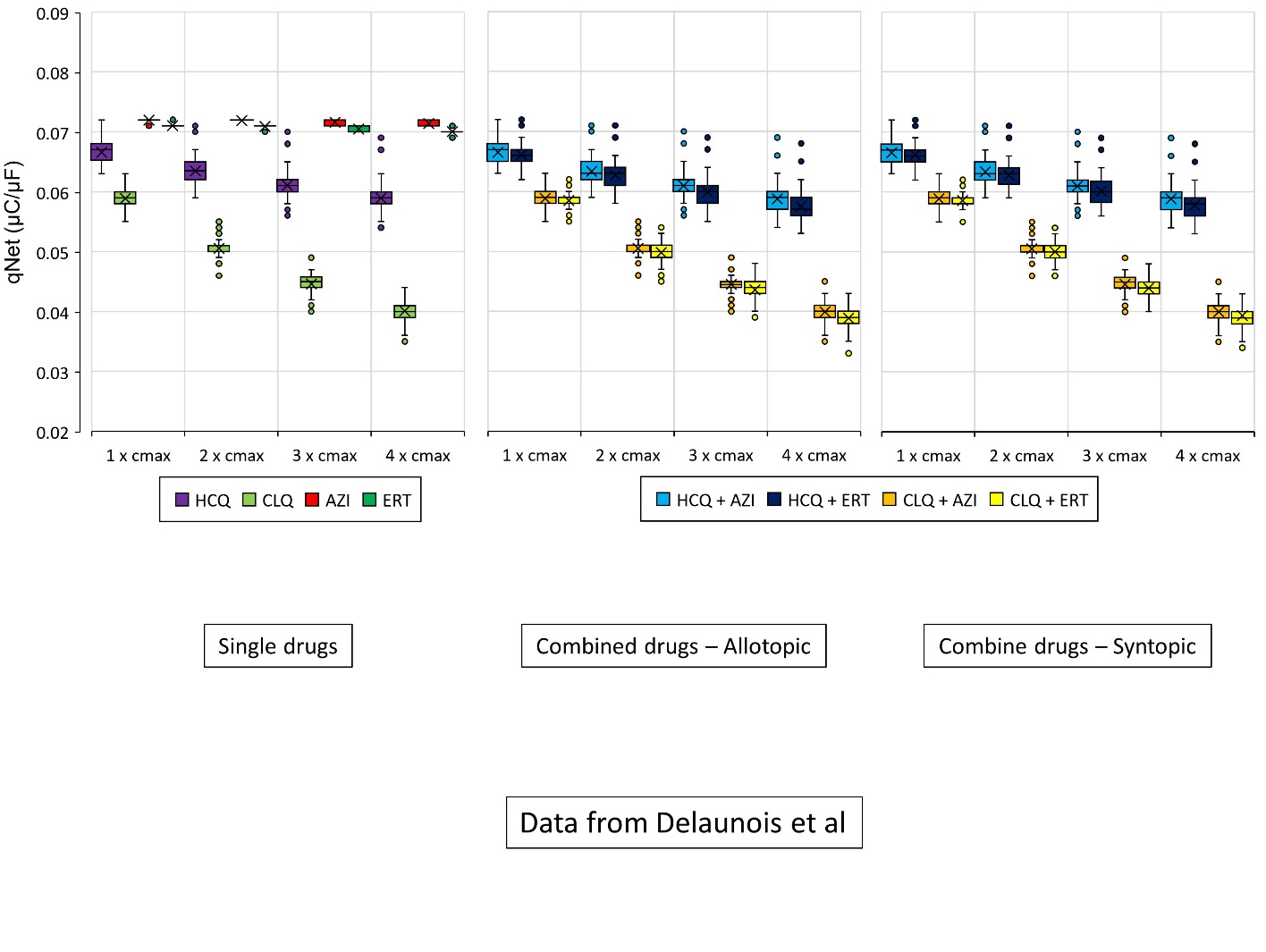

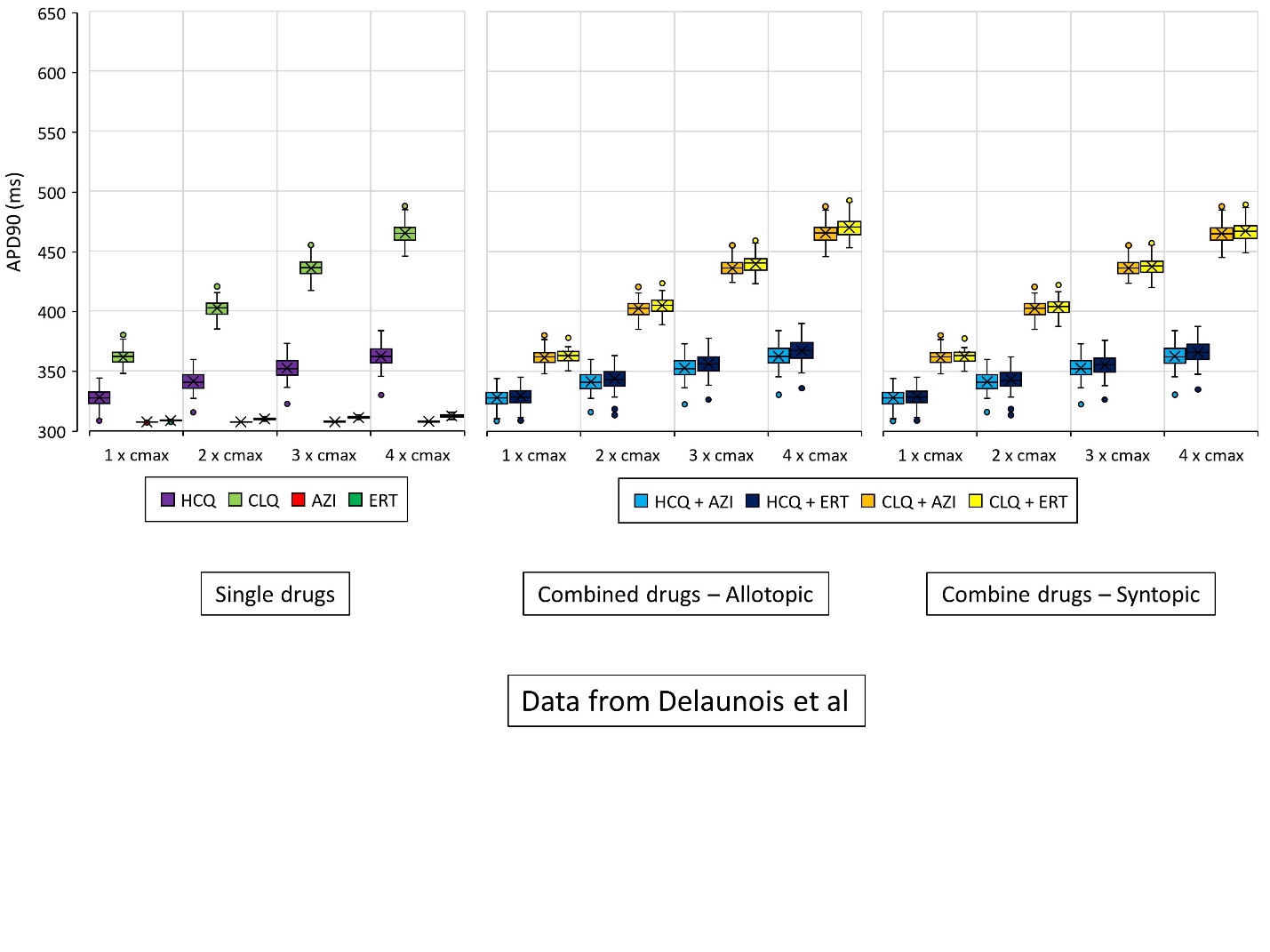


Drug concentration

Figure S2 The box plot for APD90, CaD90, and qNet as a function of drug or combined drug concentrations. The dataset incorporated in this plot is from Delaunois et al.^5^ (Table S3). The drugs in dataset are hydroxychloroquine (HCQ), chloroquine (CLQ), azithromycin (AZI), and erythromycin (ERT).

Simulation results in **Figure S2** show consistent results compared to results in Figure 2 and Figure 4 from the paper of Delaunois et al.^5^. For single drug effects, the CLQ generates the highest APD90 as a function of drug concentration followed by HCQ, ERT, and AZI, respectively. In Delaunois et al.^5^ paper, the difference between ERT and AZI is visible only in high drug concentrations. In **Figure S2**, $4\times cmax$ equals to 3,040 nM for AZI, and 13,840 nM for ERT. When comparing **Figure S2** and Figure 2 from Delaunois et al.^5^, one needs to pay attention to the actual drug concentration shown in the figure. For example, in Figure 2 of Delaunois et al.^5^ paper, the AZI at 3 $\mu M$ shows APD90 median value of around 300 ms, similar to the results in **Figure S2** for AZI at $4\times cmax$. In addition, for the combined drug effects, the combinations incorporating CQL yield higher values of APD90 compared to the combination with HCQ as shown in both **Figure S2** and Figure 2. Furthermore, for qNet values in **Figure S2**, the lowest values are generated by CLQ, followed by HCQ, ERT, and AZI, respectively, which is consistent with the results from Figure 4 of Delaunois et al.^5^ paper. CQL at $4\times cmax$ on **Figure S2** generates mean value of qNet at around 0.4 $\mu C/\mu F$ while at a similar concentration of 1.6 $\mu M$ (between 1 and 3 $\mu M$) in Figure 4 of Delaunois et al.^5^ paper, the CLQ yields median qNet value of around 0.45 $\mu C/\mu F$ at 1$\mu M$ and 0.39 $\mu C/\mu F$ at 3$\mu M$. The spread of data might be different because different population of models used in the simulation.


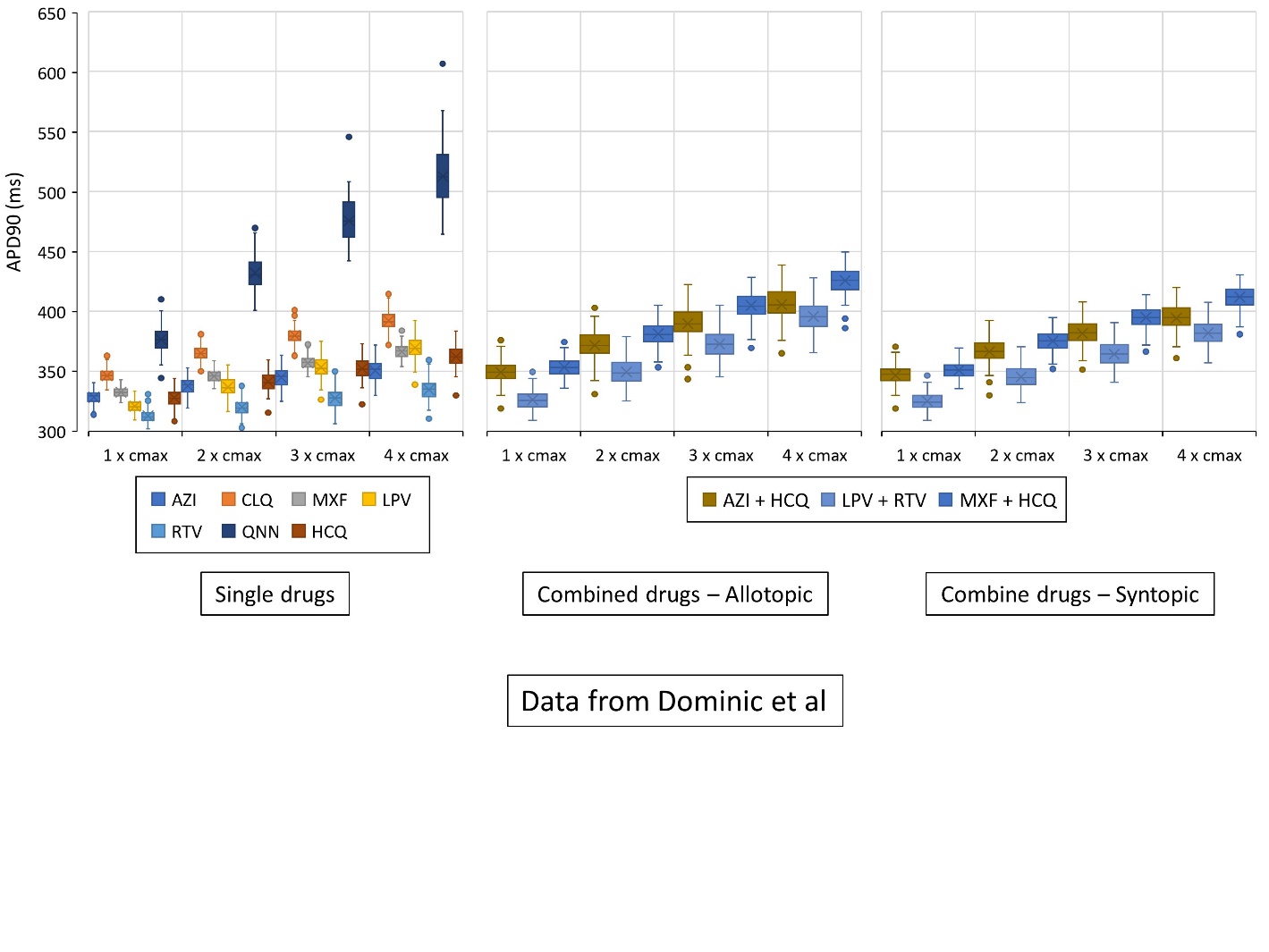


Drug concentration

**Combine drugs – Syntopic**

**Combine drugs – Allotopic**

**Single drugs**


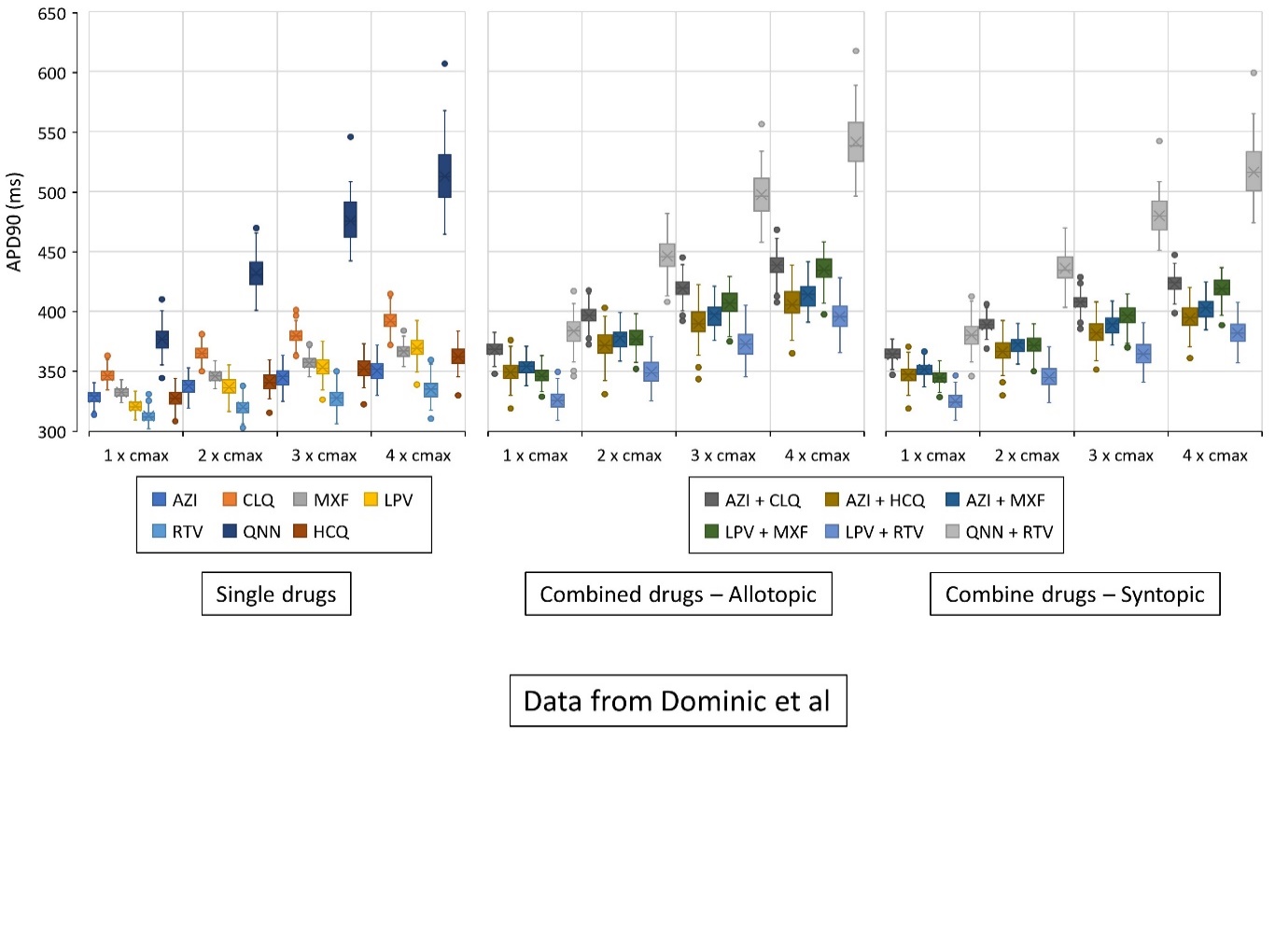

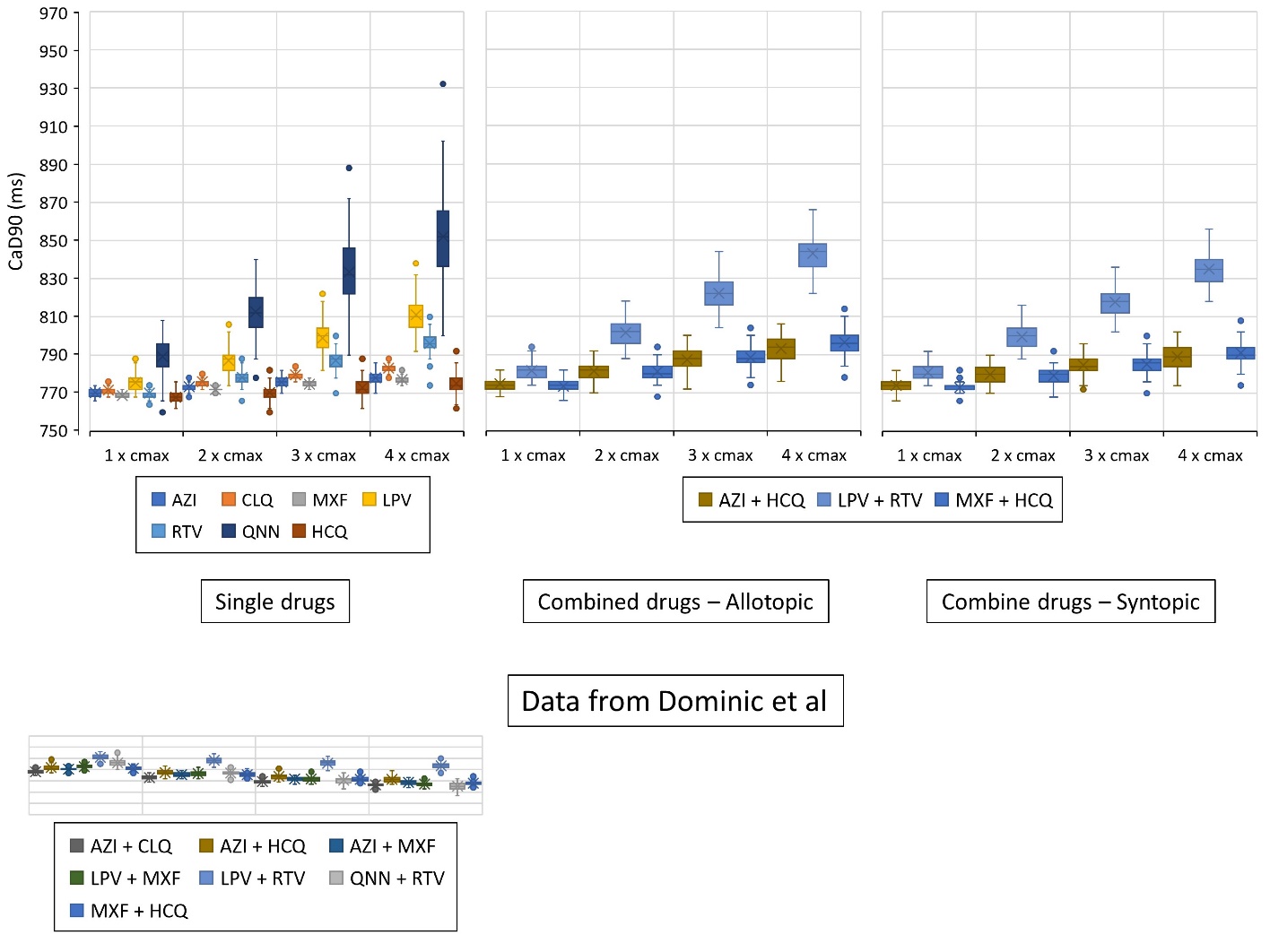

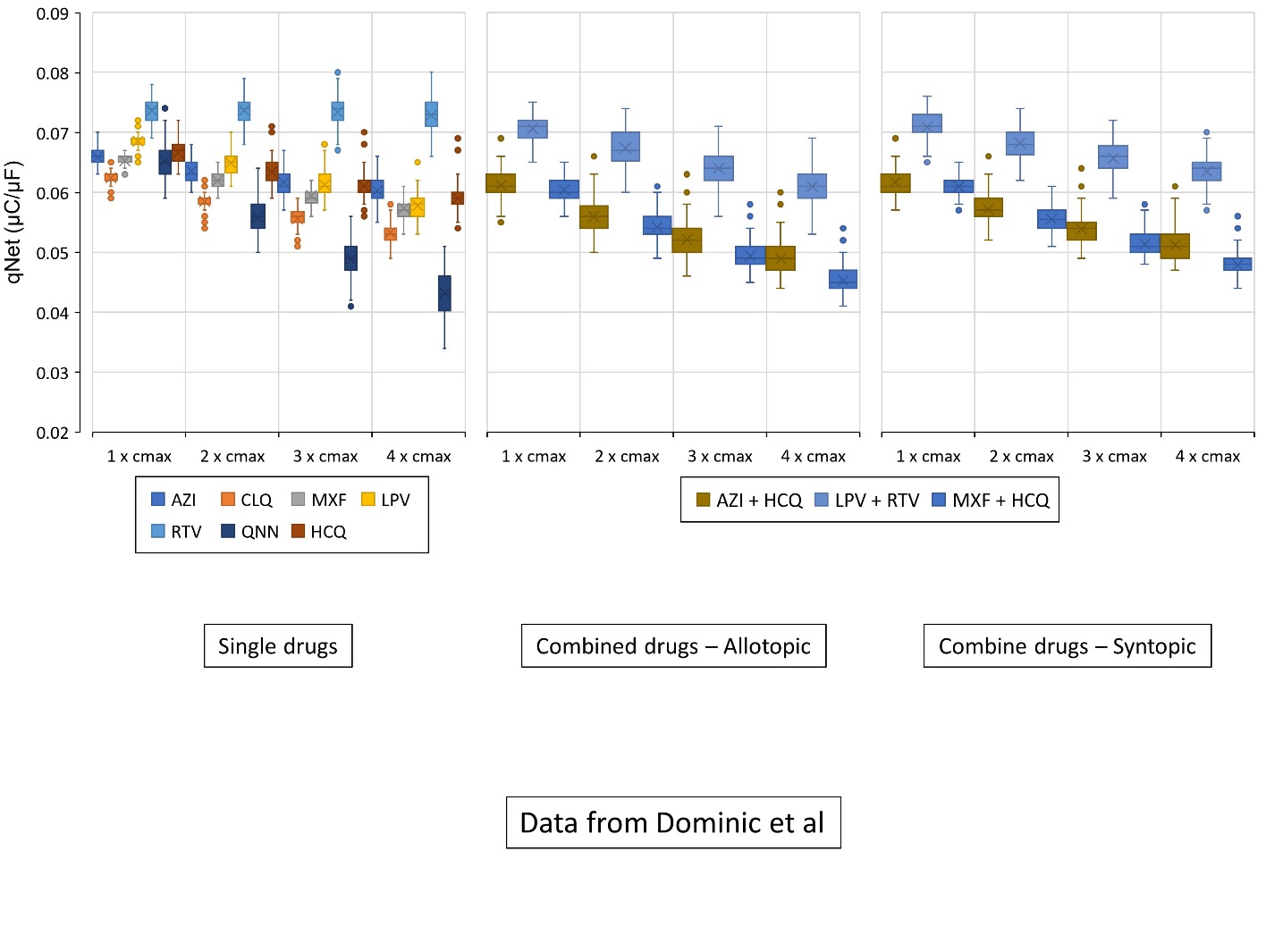

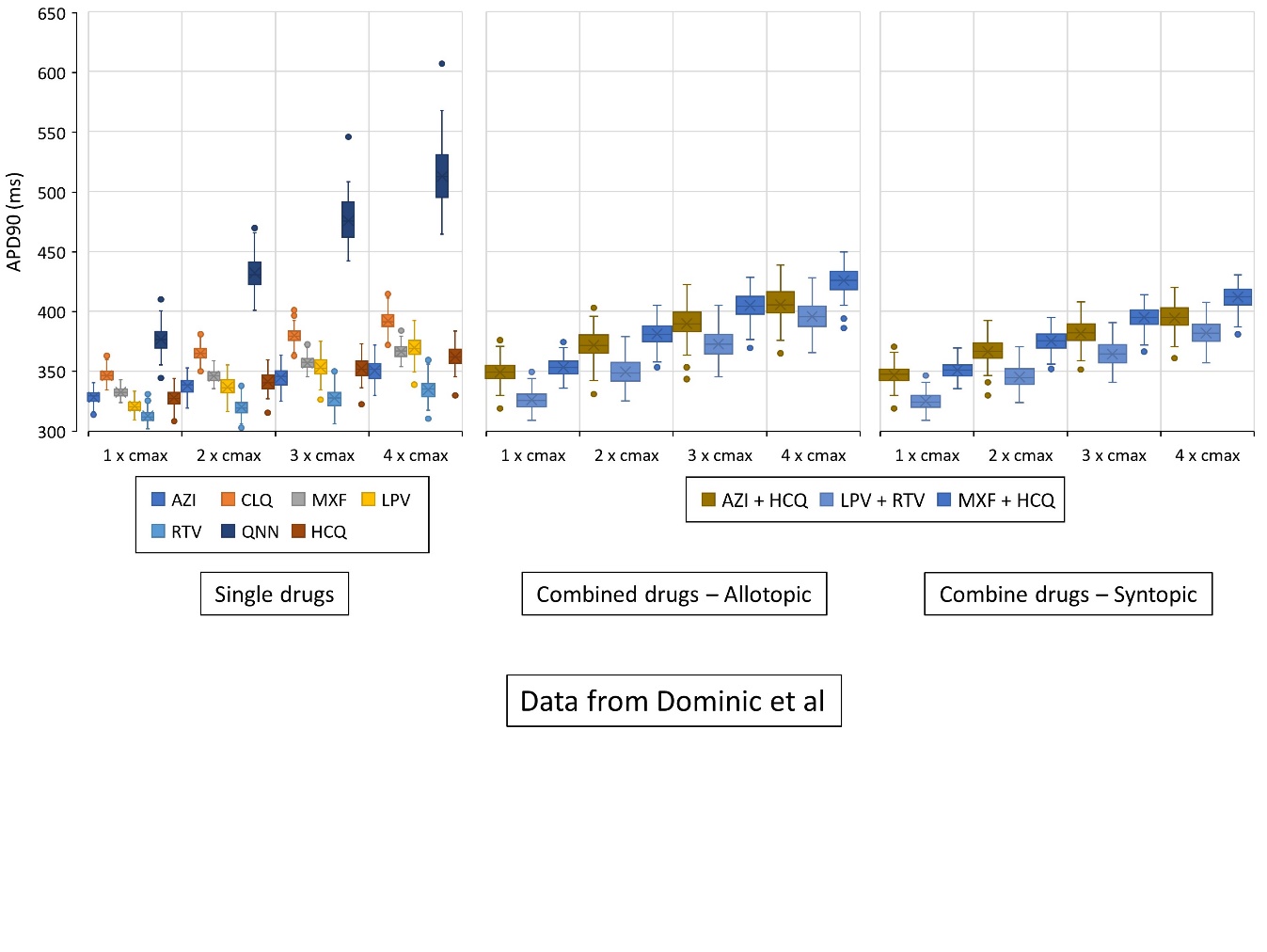


Figure S3 The box plot similar to Figure S2 with dataset similar to the work of Whittaker et al.^12^ (Table S4). The drugs in dataset are azithromycin (AZI), chloroquine (CLQ), moxifloxacin (MXF), lopinavir (LPV), ritonavir (RTV), quinine (QNN), and hydroxychloroquine (HCQ). Note that the HCQ incorporated in the simulation is from the dataset of Delaunois et al.^5^.

Results shown in **Figure S3** are also quite consistent with study reported by Whittaker et al.^12^. In **Figure S3** under single drugs and combined drugs panel, the mean value of APD90 is higher when combining AZI and HCQ compared to AZI and HCQ alone (single drug effects) that is consistent with Figure 1 panel $\left( b \right)\left( i \right)$ from Whittaker et al.^12^ paper. Combination of MXF and HCQ also produces higher mean APD90 compared to MXF and HCQ alone as shown in **Figure S3** and Figure 1 panel $\left( d \right)\left( i \right)$. For the qNet values, combination of HCQ and AZI results lower mean qNet value than that of HCQ and AZI alone. Also the same pattern observed at the combination of MXF and HCQ that it yields lower mean qNet value compared to MXF and HCQ alone.

**REFERENCES**

1. O’Hara, T., Virág, L., Varró, A. & Rudy, Y. Simulation of the Undiseased Human Cardiac Ventricular Action Potential: Model Formulation and Experimental Validation. *PLoS Comput Biol* **7**, e1002061 (2011).

2. Li, Z. *et al.* Improving the in silico assessment of proarrhythmia risk by combining hERG (Human Ether-à-go-go-Related Gene) channel-drug binding kinetics and multichannel pharmacology. *Circ Arrhythm Electrophysiol* **10**, (2017).

3. Dutta, S. *et al.* Optimization of an in silico cardiac cell model for proarrhythmia risk assessment. *Front Physiol* **8**, 616 (2017).

4. Chang, K. C. *et al.* Uncertainty quantification reveals the importance of data variability and experimental design considerations for in silico proarrhythmia risk assessment. *Front Physiol* **8**, 917 (2017).

5. Delaunois, A. *et al.* Applying the CiPA approach to evaluate cardiac proarrhythmia risk of some antimalarials used off-label in the first wave of COVID-19. *Clin Transl Sci* **14**, 1133–1146 (2021).

6. Yao, X. *et al.* In Vitro Antiviral Activity and Projection of Optimized Dosing Design of Hydroxychloroquine for the Treatment of Severe Acute Respiratory Syndrome Coronavirus 2 (SARS-CoV-2). *Clinical Infectious Diseases* **71**, 732–739 (2020).

7. Franco, B. *et al.* In Vitro Cardiovascular Effects of Dihydroartemisin-Piperaquine Combination Compared with Other Antimalarials. *Antimicrob Agents Chemother* **56**, 3261–3270 (2012).

8. Matzneller, P. *et al.* Blood, Tissue, and Intracellular Concentrations of Azithromycin during and after End of Therapy. *Antimicrob Agents Chemother* **57**, 1736–1742 (2013).

9. https://pubchem.ncbi.nlm.nih.gov/.

10. Krasniqi, S. *et al.* Blood, Tissue, and Intracellular Concentrations of Erythromycin and Its Metabolite Anhydroerythromycin during and after Therapy. *Antimicrob Agents Chemother* **56**, 1059–1064 (2012).

11. Crumb, W. J., Vicente, J., Johannesen, L. & Strauss, D. G. An evaluation of 30 clinical drugs against the comprehensive in vitro proarrhythmia assay (CiPA) proposed ion channel panel. *J Pharmacol Toxicol Methods* **81**, 251–262 (2016).

12. Whittaker, D. G. *et al.* Cardiac TdP risk stratification modelling of anti-infective compounds including chloroquine and hydroxychloroquine. *R Soc Open Sci* **8**, 210235 (2022).
